# Dual leucine zipper kinase is necessary for retinal ganglion cell axonal regeneration in *Xenopus laevis*

**DOI:** 10.1093/pnasnexus/pgad109

**Published:** 2023-03-30

**Authors:** Lindsay Fague, Nicholas Marsh-Armstrong

**Affiliations:** Department of Ophthalmology and Vision Science, UC Davis Eye Center, University of California, Davis, 1275 Med Science Drive Rm. 3451, Davis, CA 95616, USA; Department of Ophthalmology and Vision Science, UC Davis Eye Center, University of California, Davis, 1275 Med Science Drive Rm. 3451, Davis, CA 95616, USA

**Keywords:** regeneration, *Xenopus*, optic nerve, retina

## Abstract

Retinal ganglion cell (RGC) axons of the African clawed frog, *Xenopus laevis*, unlike those of mammals, are capable of regeneration and functional reinnervation of central brain targets following injury. Here, we describe a tadpole optic nerve crush (ONC) procedure and assessments of brain reinnervation based on live imaging of RGC-specific transgenes which, when paired with CRISPR/Cas9 injections at the one-cell stage, can be used to assess the function of regeneration-associated genes in vivo in F0 animals. Using this assay, we find that map3k12, also known as dual leucine zipper kinase (Dlk), is necessary for RGC axonal regeneration and acts in a dose-dependent manner. Loss of Dlk does not affect RGC innervation of the brain during development or visually driven behavior but does block both axonal regeneration and functional vision restoration after ONC. Dlk loss does not alter the acute changes in mitochondrial movement that occur within RGC axons hours after ONC but does completely block the phosphorylation and nuclear translocation of the transcription factor Jun within RGCs days after ONC; yet, Jun is dispensable for reinnervation. These results demonstrate that in a species fully capable of regenerating its RGC axons, Dlk is essential for the axonal injury signal to reach the nucleus but may affect regeneration through a different pathway than by which it signals in mammalian RGCs.

**Significance Statement**

In mammals, damage to retinal ganglion cell (RGC) axons results in neuronal death and irreversible blindness. Most interventions in mice produce only modest regeneration with little vision restoration. Therefore, learning how other vertebrates regenerate their RGC axons is of great interest. We report new assays in one such species, *Xenopus laevis*, which enable live imaging of axon regeneration and scalable interrogation of gene function via CRISPR/Cas9. We find that dual leucine zipper kinase (*dlk*) is essential for RGC axon regrowth and vision restoration. However, the downstream transcription factor Jun seems dispensable for regeneration. Thus, though Dlk seems to assist in conveying an injury signal back to the soma, it may act by different mechanisms in frog RGCs than in mammals.

## Introduction

In mammals, the central nervous system (CNS) is incapable of productive axonal regeneration. Injury to axons invariably results in not only the rapid degeneration of the injured axons but also most often subsequent cell death. As the visual system is part of the CNS, optic neuropathies that affect the axons of retinal ganglion cells (RGCs), the sole projecting neurons of the eye, eventually result in partial or complete irreversible vision loss. In acute situations of traumatic brain injury or ischemic optic neuropathies, this vision loss can occur quickly, over days or weeks (1–3). In more chronic optic neuropathies such as glaucoma, the leading cause of irreversible blindness worldwide, insult to RGC axons is more focal and asynchronous. This results in vision loss that is asymmetric and progressive, unfolding over years or decades (4, 5).

In contrast to mammals, many nonamniotic vertebrates possess the ability to regenerate injured RGC axons, successfully reinnervate appropriate brain targets, and regain functional vision. Roger Sperry's classic eye rotation experiments in newts (6) and forced nerve uncrossing experiments in anuran amphibian species (7) long ago showed that in these vertebrates, fully disconnected RGC axons are able to reconnect and drive visually driven behaviors. Given that the genome of *Xenopus laevis* bears significant sequence similarity to that of humans (79% of disease-causing genes in humans have clear homologs in *Xenopus* (8, 9)), it is likely that at least some of the molecular pathways responsible for the RGC axonal regeneration of anurans remain extant in the human genome. As such, understanding which genes and pathways are essential for successful RGC axonal regeneration in *X. laevis*, and which are not, may provide critical information for future attainment of RGC axonal regeneration in clinical settings.

Here, we have developed an optic nerve crush (ONC) model in young *X. laevis* tadpoles that is suitable to query genes involved in RGC axonal regeneration via CRISPR-based loss-of-function studies. Using these assays, we show that a gene previously known to be important in the response of neurons to injury, dual leucine zipper kinase (*dlk*), is essential for RGC axonal regrowth and for the restoration of vision after injury. The actions of Dlk are thought to act largely through a MAPK cascade that ultimately phosphorylates the transcription factor Jun, leading to its nuclear translocation and consequent large transcriptional changes. We find that Dlk functions largely cell autonomously within RGCs and that Dlk loss does not impair axon growth by noninjured RGCs, only blocking the regeneration of the damaged RGC axons. We further find that while ONC affects mitochondrial movement along RGC axons soon after axon injury, Dlk loss has no measurable effect on this acute injury response. Finally, Dlk loss eliminates the injury-induced phosphorylation and nuclear translocation of the transcription factor Jun that occurs days later within RGCs and does so in a dose-dependent manner that parallels the dose-dependent effect on regeneration, and yet Jun itself is not required for regeneration. As such, our results suggest that Dlk acts within RGCs across vertebrates in communicating the axon injury signal back to the RGC soma. However, the determination whether the RGCs will regenerate their axons is clearly downstream of Dlk, is surprisingly dose dependent, and does not appear to require Jun-dependent transcriptional changes.

## Results

### A tadpole ONC model

**Fig. 1.**
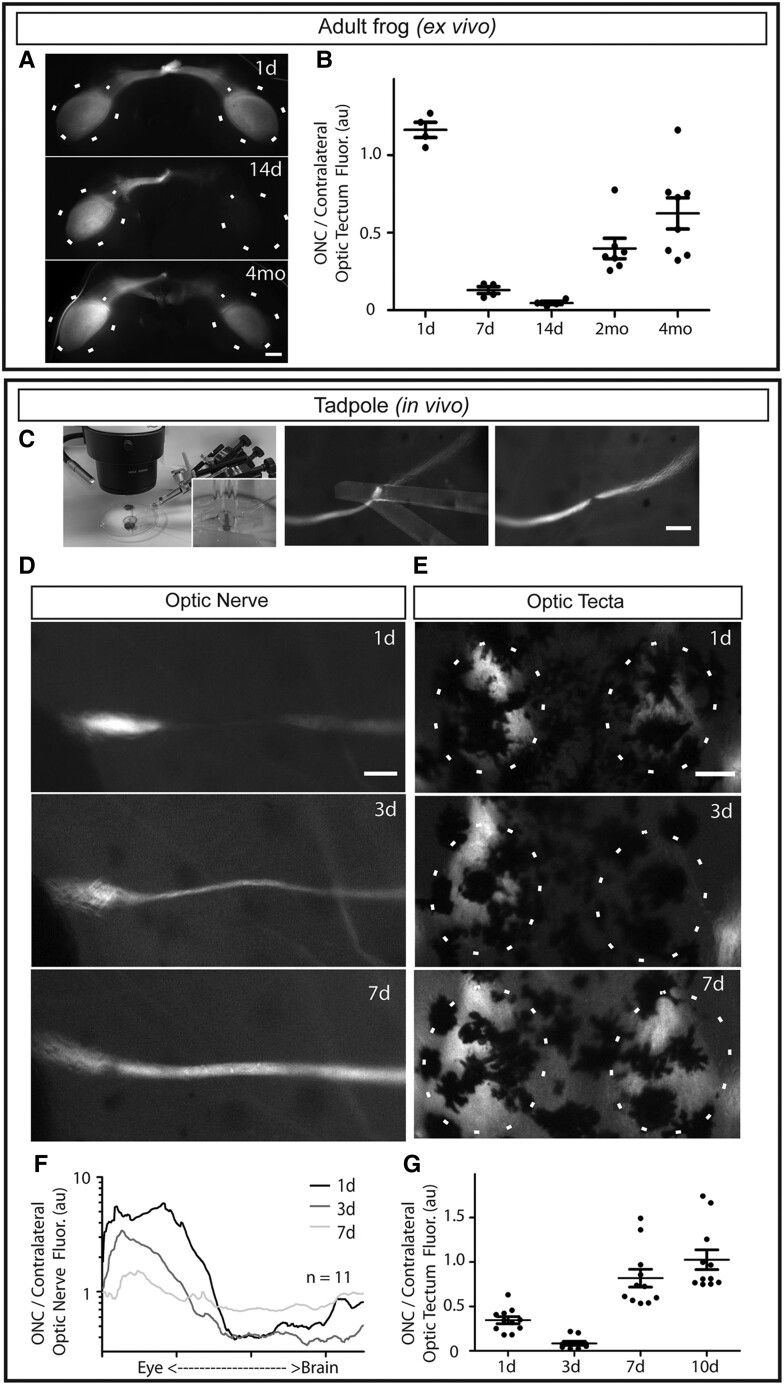
ONC assay in young *Xenopus laevis* tadpoles has a fast time course and enables live imaging of degeneration and regeneration in optic nerves and optic tecta. A and B) In adults, regeneration takes months and must be measured ex vivo. A) Dissected flattened brain preparations showing GFP driven by an RGC-specific promoter (the fourth month image shows what appears to be a doubled optic chiasm due to a dissection artifact). Scale bar = 1 mm. B) Time course of regeneration in adult frogs. C–G) Novel surgical and live-imaging/quantification assays in young tadpoles. C) Young tadpole ONC surgical procedure. Micromanipulator-mounted needles, visualized alongside fluorescent optic nerves, are used to crush the optic nerve in 8-day-old tadpoles. (Contrast settings for the needles in the middle panel were nonuniformly lightened to better show their placement.) D and E) The optic nerve and optic tecta of the same animal are live imaged over the course of axonal degeneration and regeneration. Scale bar = 100 μm. F) Measures of fluorescence along the injured optic nerve, normalized to equivalent positions along the uninjured contralateral nerve, show the transient large increase in fluorescence proximal to the injury (note the logarithmic scale) and that regeneration of axons is largely complete by 7 days post-ONC. G) Measures of fluorescence in crushed optic tecta relative to contralateral tecta similarly show that denervation is complete by 3 days and innervation is largely restored by 7 days post-ONC.

The optic nerve was crushed in adult *X. laevis* as previously done by us and others (10–12) but in transgenic animals where the RGCs express a membrane-localized GFP under the control of the RGC-specific zebrafish Isl2b promoter (Isl2b:mem-GFP) (13). Comparison of the fluorescence intensity in the tecta innervated by the crushed nerve to that in the contralateral (uninjured) tecta in the same animals enables rapid estimation of the extent of denervation and reinnervation (14). In contrast to the lateral geniculate nucleus which receives binocular input starting at metamorphosis (15), RGC innervation of the optic tecta in *X. laevis* at this stage is largely if not exclusively monocular. In adult frogs, where the dermis is opaque and the brain is fully encased in the skull, an ex vivo preparation must be used to visualize the optic tecta, here done in dissected partially hemisected and flattened brains (Fig. 1A). Using the relative (injured/contralateral) fluorescence measure, we find that the injured optic tectum becomes completely denervated by 14 days post-ONC and only becomes largely reinnervated 2–4 months later (Fig. 1B). Notably, even by 4 months post-ONC, the tectum innervated by the injured nerve displays a lower fluorescence intensity compared with the contralateral tectum, similar to what we previously showed using a cytoplasmic GFP reporter, where it took 7 months to reach near-full tectal reinnervation (12).

Since RGC axonal regeneration in adult frogs is slow and must be assessed ex vivo, we sought to develop an assay better suited for timely functional interrogation of genes involved in RGC axonal regeneration. To this end, we designed an ONC technique that can be performed on 8-day-old transgenic *X. laevis* tadpoles. Micromanipulator-mounted glass needles, visualized under a fluorescence stereomicroscope, are used to create a highly focal and reproducible ONC injury to fluorescent optic nerves (Fig. 1C). Given the transparent dermis and relatively few melanophores of young *X. laevis* tadpoles, this preparation allows for repeated in vivo imaging of the optic nerves and tecta within individual animals. Comparing the fluorescence intensities of the injured versus contralateral tecta (Fig. 1E and G) revealed that both denervation and reinnervation were much faster than in adults; denervation is completed by 3 days and reinnervation is near maximal by 7 days post-ONC. To measure axonal degeneration and regeneration in the optic nerves, images of both crushed and contralateral nerves were delineated and scaled to equal lengths, and then the fluorescence across the length of the crushed nerve was normalized to that at equivalent positions along the contralateral nerve (Fig. 1D and F). Consistent with the tectal measures, the optic nerve measures showed axonal degeneration distal to the injury site to be maximal at 3 days and axonal regrowth near completion by 7 days post-ONC. At 1 day post-ONC, axonal degeneration is largely confined near the crush, while by 3 days post-ONC, the distal portions of RGC axons have been largely removed, presumably by Wallerian degeneration (15). Notably, as soon as 1 day post-ONC and still evident at 3 days post-ONC, there is a prominent increase in fluorescence proximal to the site of injury (nearer to the eye), which likely represents the retraction of injured axons toward the soma, a phenomenon commonly observed after axonal injuries (16, 17). Thus, our assay provides a model to study vertebrate RGC axon degeneration and regeneration which compares favorably with other models in that it takes place over just 1 week, individual animals can be followed over time, and the imaging and quantification are relatively quick and easy.

### Tadpole ONC induces little to no RGC death and tectal fluorescence measures report mainly axonal regeneration

**Fig. 2.**
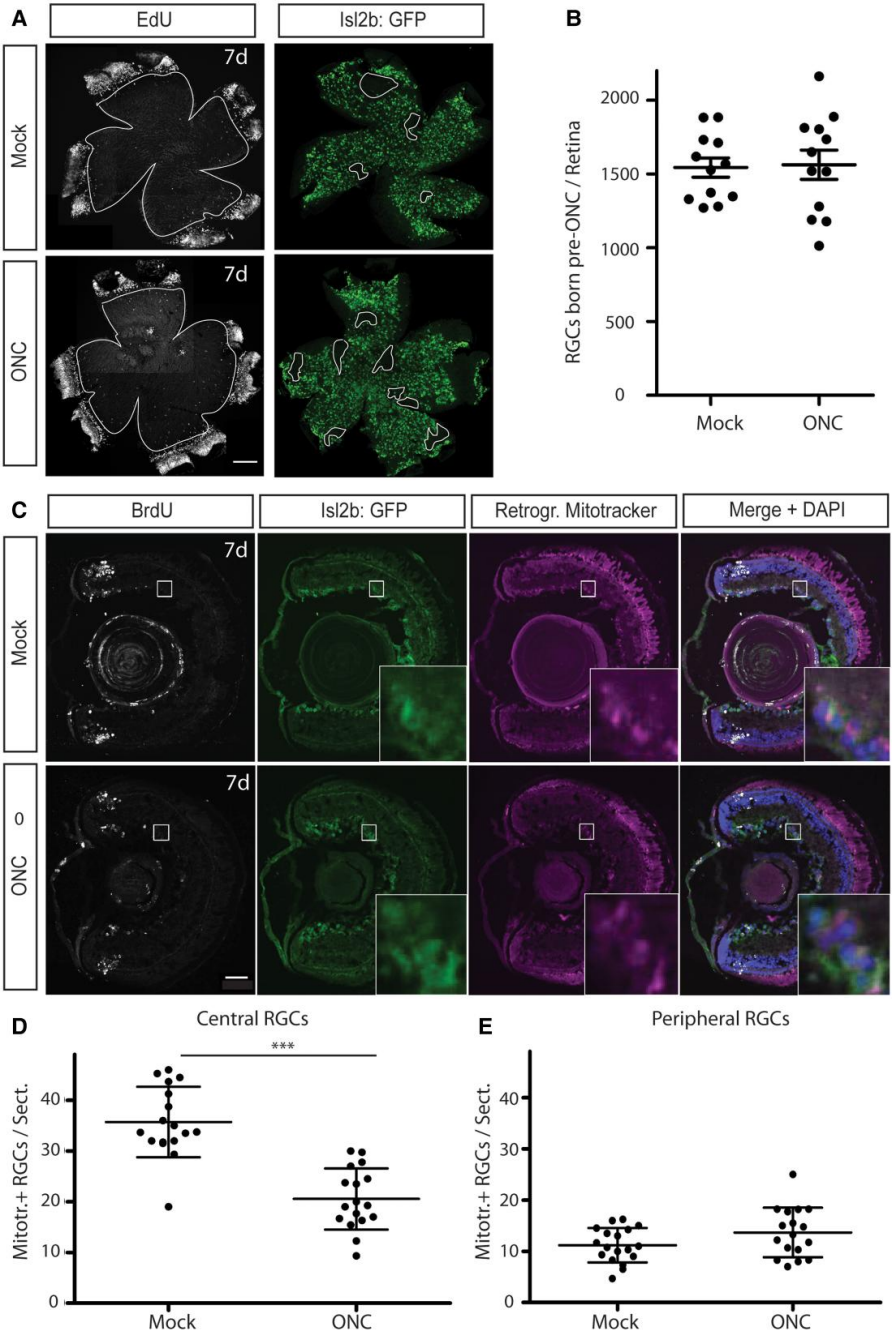
In young tadpoles, the RGCs whose axons were crushed do not die and provide the majority of the tectal innervation 7 days post-ONC. A and B) RGCs present in the retina prior to the ONC survive and remain at numbers similar to mock-crushed retinas 7 days post-ONC. A) An EdU pulse administered on the day of ONC to tadpoles that express cytoplasmic GFP in their RGCs distinguishes central RGCs born prior to ONC from peripheral RGCs born after ONC. (Contours in the GFP images show dissection artifacts that were excluded from the RGC automatic counting.) Scale bar = 100 μm. B) Automated cell counts of central RGC numbers show that by 7 days post-ONC, there is no significant death of the injured RGCs. *N* = 12 retinas each for mock and ONC retinas. C) BrdU labeling on the day of ONC to delineate RGCs born prior to ONC from RGCs born after ONC is combined with retrograde tracing by Mitotracker at 6 days post-ONC to label those RGCs whose axons have reached the optic tectum. Merge includes nuclear labeling with DAPI. Scale bar = 50 μm. D and E) Manual counts of those RGC soma which are labeled 24 h after retrograde tracer application find that the majority of the tectal innervation 7 days post-ONC derives from the injured central RGCs. *N* = 15 mock and 18 ONC retinas, with a minimum of 4 cryosections counted per retina. ***P < 0.001.

In adult *X. laevis*, it has been reported that up to 20% of RGCs die in the first weeks following ONC (18), similar to adult zebrafish (19). Furthermore, the retinas of *X. laevis* continuously grow at their periphery by the proliferation and differentiation of retinal progenitors at the ciliary marginal zone (CMZ), also similar to zebrafish (20–22). This CMZ-based generation of all retinal cell types, including RGCs, occurs throughout the animal’s lifetime but is most robust at premetamorphic stages (23). Thus, it was possible that tectal innervation after ONC in the young tadpoles might not be due to axonal regeneration from the axons of crushed RGCs but instead derive solely from the new RGCs born at the CMZ after injury. To determine whether there was significant RGC death post-ONC, we administered an EdU pulse just after ONC or mock injury in *X. laevis* tadpoles whose RGCs expressed cytoplasmic GFP (Isl2b:GFP, previously described in Watson *et al*. (12)), as this transgene produces sufficiently discrete visualization of soma to enable automatic counting of RGCs. Flatmounts of the EdU-labeled retinas enabled clear demarcation of the RGC cells interior to the EdU pulse, which represent the RGCs born prior to ONC and whose axons would have been injured by the ONC (Fig. 2A). Cell counts using a previously validated algorithm (24) found no significant difference in numbers of RGCs central to the EdU pulse between ONC and mock-crushed retinas (Fig. 2B), showing that in these young tadpoles most, if not all, RGCs survive the axonal injury.

A more critical question was whether the majority of the tectal innervation observed at 7 days post-ONC came from the axonal regeneration of the RGGs disconnected from the brain by ONC or from the newly born RGCs whose axons were innervating the tectum for the first time. To answer this question, we administered a BrdU pulse alongside either ONC or mock crush to Isl2b:GFP transgenic tadpoles at 8 days postfertilization (dpf), then followed this 6 days later (14 dpf) by insertion of a small Mitotracker-soaked piece of Gelfoam into the injured optic tectum to retrogradely label the RGCs that had successfully innervated the optic tectum. These retinas were analyzed in sections rather than wholemounts as the peripheral-most RGCs are difficult to visualize in wholemounts due to the curling of the flatmounted retinas (Fig. 2C). When we quantified the number of RGC soma labeled by Mitotracker which were colocalized with or peripheral to the BrdU labeling (representing RGCs born after ONC), we found no significant difference in numbers between ONC or mock-crushed retinas (Fig. 2E), showing that RGC axon injury does not affect either the generation or brain innervation by new RGCs derived from the CMZ. Importantly, in both the retinas subjected to ONC and those subjected to mock crush, the majority of the RGCs that were connected to the optic tectum were central to the BrdU pulse, demonstrating that the injured RGCs not only survive but successfully re-establish connections with the optic tectum. There were fewer retrogradely labeled RGC soma interior to the BrdU pulse in ONC retinas compared with mock-crushed retinas (Fig. 2D), suggesting that either some injured RGCs do not reconnect with the brain or that the regeneration of RGC axons may take longer than innervation of the tectum by newly born RGCs. Thus, innervation of the optic tectum after ONC in young tadpoles involves a significant contribution from newly born RGCs, but the majority of the innervation derives from the crushed RGC axons, thus making ONC in young tadpoles suitable for the interrogation of RGC axonal regeneration genes.

### Dlk functions in tectal reinnervation after ONC

**Fig. 3.**
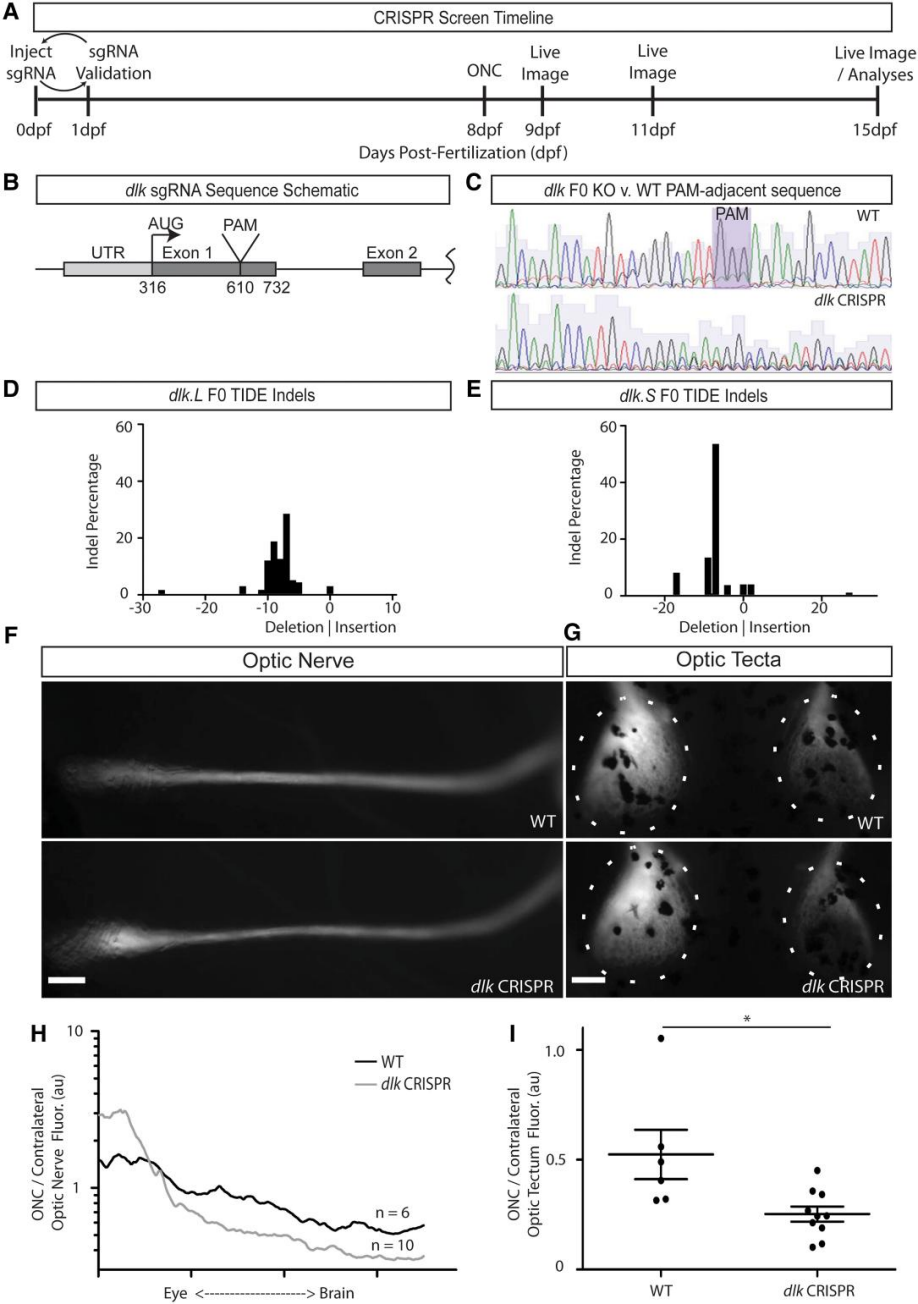
F0 CRISPR screen reveals that Dlk is involved in RGC axon tectal innervation after ONC. A) Timeline for F0 CRISPR screen. F0 KO animals were generated using a transgenic line in which RGCs express GFP. sgRNA + Cas9 protein was injected within 30 min of fertilization. One day later, PCR genotyping was performed using genomic DNA from pools of 5 embryos to assess KO efficiency by TIDE analyses. At 8 days post-fertilization, GFP+ tadpoles were subjected to monocular ONC, followed by 3 days of imaging (1 day, 3 days, and 7 days post-ONC) and then tissue harvesting. B) Schematic of the first 2 exons of *dlk* showing the location of the sgRNA PAM within exon 1. C) Sample sequencing traces from uninjected (top) and *dlk* gRNA injected (bottom) embryos near the predicted CRISPR cut site. D and E) *dlk* gRNA F0 indel efficiency was >90% for both *dlk.S* and *dlk.L* alleles. F and G) Representative live images at 6 days post-ONC of animals near the mean of the groups (in some *dlk* gRNA–injected animals, the phenotype was far more pronounced). Optic nerve and optic tecta are from the same animals. F) *dlk* gRNA–injected animals have a nerve phenotype consistent with an inhibition or delay of RGC axon growth after ONC: thinner ON distally and thicker ON proximally relative to the ONC site. Scale bar = 100 μm. G) *dlk* gRNA–injected animals have somewhat diminished tectal innervation after ONC. H) Measures of fluorescence across the crushed nerve normalized to equivalent positions along the uninjured contralateral nerve (mean of 6 and 10 for WT and *dlk* gRNA–injected animals, respectively) show that the nerves of *dlk* gRNA–injected animals at 6 days post-ONC have a profile consistent with an inhibition or delay of RGC axon growth after ONC. I) Measures of fluorescence comparing tectal fluorescence in injured tecta denervated after ONC to contralateral tecta in the same animals show that *dlk* gRNA–injected animals have significantly decreased tectal innervation. *P < 0.05.

Diverse cell-intrinsic and -extrinsic factors have been found to contribute to RGC axonal regeneration in mammals (25), but which are most important in the regeneration-capable RGCs of *X. laevis* is unknown. To investigate the effect of individual genes on RGC axonal regeneration in young tadpoles, we employed a CRISPR/Cas9-based approach using the progeny of Isl2b:mem-GFP transgenic frogs (Fig. 3A). To produce partial loss-of-function animals, CRISPR/Cas9 injection was carried out at the one-cell stage similarly to what has been previously described (26, 27); the efficiency of the guides was validated at 1 day postinjection using TIDE (Tracking of Indels by Decomposition) (28), which compares the mixed sequence traces derived from the CRISPR/Cas9-injected animals to the nearly homogenous sequence trace derived from noninjected animals (Fig. 3B and C). We set a minimum threshold of 80% overall indels and 50% frameshift indels for sgRNAs to be sufficiently efficient to progress into the tadpole ONC assay. Once we found an sgRNA that exceeded this threshold, we performed unilateral ONC at 8 dpf, followed by live imaging at 1, 3, and 6/7 days post-ONC to measure tectal denervation and reinnervation; the final measure depended on when the control group had reached approximately half the tectal innervation in the crushed tectum when compared with that observed contralaterally. Thus, from microinjection to assessment of denervation and reinnervation, the testing time for a new sgRNA was ∼15 days.

One of the first genes tested was *Mitogen-activated protein kinase kinase kinase 12* (*map3k12*), also known as *dual leucine zipper kinase or dlk*. Dlk was chosen because it functions in a mitogen-activated protein kinase pathway upstream of Jun N-terminal kinases, cell-injury signals (29–34) that in turn phosphorylate the transcription factor Jun (32), which in neurons mainly leads to either apoptosis, as in mammalian CNS neurons including RGCs, or axonal regeneration, as in invertebrates or the PNS neurons of mammals. After screening multiple sgRNAs, the one chosen to proceed to the ONC assay at 8 dpf targets the first coding exon of *dlk* (Fig. 3B) and was shown to produce 90% mutation frequency near the target site (Fig. 3C), with >50% being frameshift mutations in both S and L chromosomes (Fig. 3D and E). A −7 deletion was the most common allele on both chromosomes, but highly varied in-frame and out-of-frame insertions and deletions were produced. When transgenic Isl2b:mem-GFP *dlk* sgRNA-injected F0 animals were subjected to ONC and then analyzed at 6 days post-ONC, on average these animals displayed significantly decreased tectal reinnervation compared with uninjected WT control embryos from the same in vitro fertilization (Fig. 3G and I). Furthermore, both the raw images and the crushed/contralateral nerve fluorescence measures (Fig. 3F and H) showed that the *dlk* sgRNA–injected animals at 6 days post-ONC manifested the proximal nerve thickening observed in WT animals at 3 days post-ONC (Fig. 3F), characteristic of when axons have degenerated but not yet begun to regenerate. Thus, both the qualitative nerve assessments and the quantitative optic tectum measures in the F0 injected animals suggested that Dlk is to some degree required for RGC axonal regeneration in *X. laevis*.

**Fig. 4.**
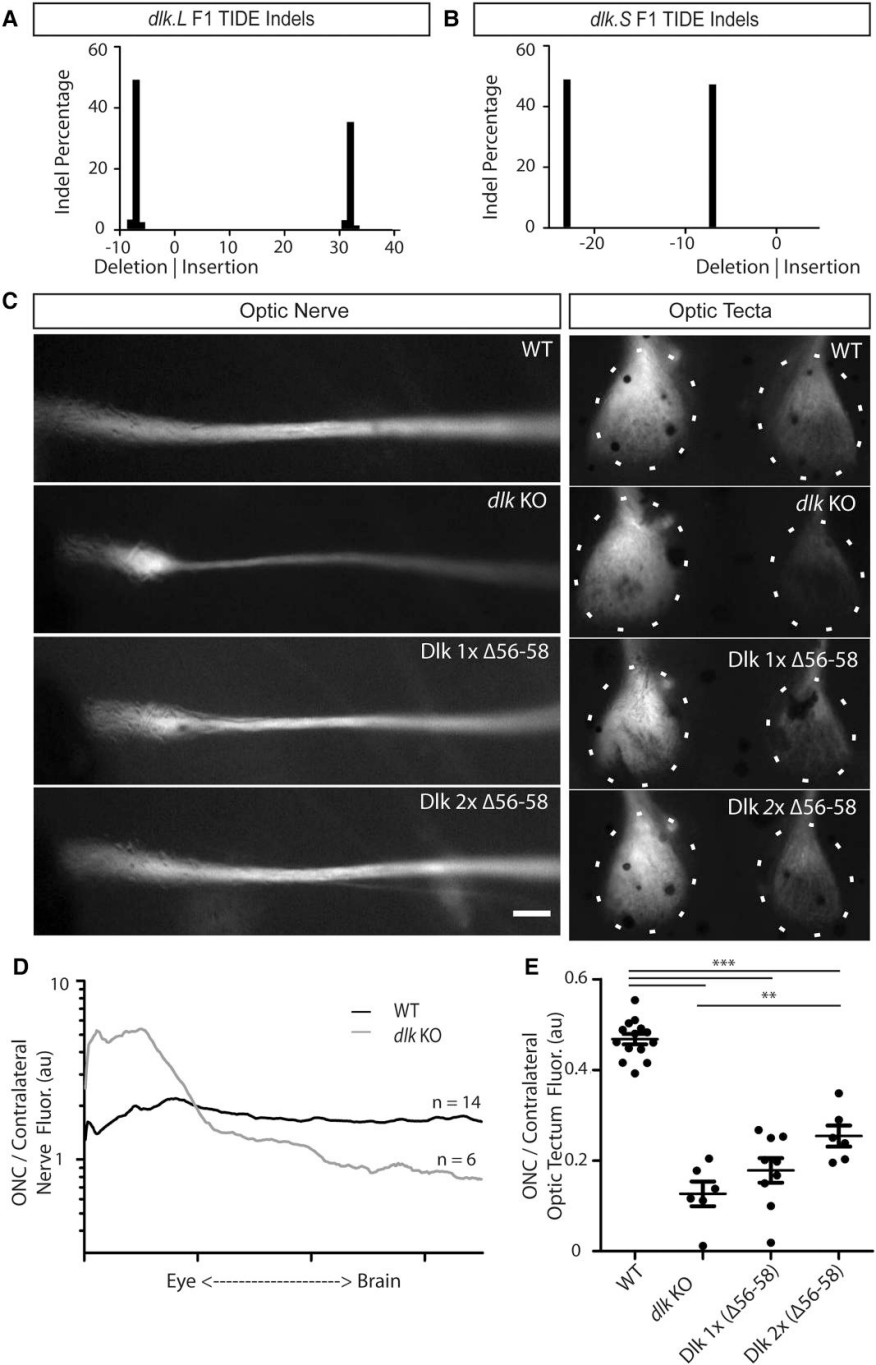
Analyses of F1 animals derived from the original F0 screen demonstrate that the effect of Dlk on RGC-axonal regeneration is dose dependent. A and B) Example TIDE results showing dual frameshift mutations in the S and L chromosomes. A) TIDE trace from an F1 animal carrying a −7 deletion and a +32 insertion in its L chromosome. B) TIDE trace from a different F1 animal carrying −23 and −7 deletions in its S chromosome. C) In one line, a small in-frame deletion that eliminates 3 amino acids, Dlk Δ56–58, occurred in both S and L chromosomes of F1 animals. Two copies of Dlk Δ56–58 resulted in the least severe phenotype, while having only frameshift alleles (*dlk* KO) resulted in the most severe phenotype. Scale bar = 100 μm. D) Measures of fluorescence across the crushed nerve normalized to equivalent positions along the uninjured contralateral nerve show that, compared with WT crushed nerves, in F1 *dlk* full KO animals, the proximal nerve remains enlarged, while the distal nerve fluorescence is attenuated at 6 days post-ONC. E) Measures of fluorescence comparing the crushed to the uninjured contralateral optic tecta show an allelic series in which rising copy number of the Dlk Δ56–58 mutation results in progressively less severe axonal regeneration defects. ***P < 0.001, **P < 0.01.

To determine the extent of the effect of Dlk deletion on regeneration and to examine the mechanism by which Dlk acts, we raised gRNA-injected animals to sexual maturity and repeated the tadpole ONC assay in F1 progeny created by breeding together two *dlk* gRNA–injected animals carrying high mutation frequencies, one of which also carried the Isl2:mem-GFP transgene. Because multiple cells contribute to the germline and sgRNA/Cas9 injection at the one-cell stage results in mosaic animals, and because *X. laevis* is allotetraploid, F1 animals could carry as many as four different *dlk* alleles with different combinations in different siblings, as was confirmed by TIDE analyses (Fig. 4A and B). Consequently, analyses of regeneration using F1 tadpoles required genotyping of every animal to identify those carrying only frameshift alleles (hereafter referred to as *dlk* knockout [KO]). An advantage of such an approach is that it can also reveal dosage effects. We bred two founders which carried no WT alleles and a high frequency of frameshift alleles and obtained animals which genotyping revealed were an allelic series: carrying either only frameshift mutations in *dlk* on both chromosomes, 1 copy (L chromosome) of an in-frame *dlk* −9 mutation, predicted to delete amino acids 56 to 58 (Dlk Δ56–58), or 2 copies (S and L chromosomes) of this Dlk Δ56–58 allele (Fig. 4E). While the thicker proximal nerve phenotype was observed in all groups carrying *dlk* mutations, the more quantitative tectal innervation measures showed the degree of reinnervation varied depending on Dlk dosage. Tadpoles carrying only frameshift mutations, and which therefore presumably possess no functional Dlk, showed the most severe tectal reinnervation defects as well as the most severe nerve phenotypes, while those with 1 or 2 copies of Dlk Δ56–58 had intermediate phenotypes (Fig. 4C–E). To confirm this dosage dependence in a different way, we used separate founders and compared animals with four WT *dlk* alleles to ones that carried two WT and two frameshift alleles. Here, 50% loss of Dlk also resulted in an inhibition of tectal innervation after ONC (Fig. S1), further supporting a dosage-dependent effect of Dlk on RGC axonal regeneration.

### Dlk likely functions autonomously within *X. laevis* RGCs

Because the CRISPR injection and F0 interbreeding process result in global KOs, it was possible that the effect of Dlk loss on RGC regeneration was not cell autonomous and instead was the result of perturbing some Dlk-dependent function in neighboring cells. Oligodendrocyte, astrocyte, or resident macrophage (microglia) populations within the optic nerve, all of which would also be devoid of Dlk in our KO tadpoles, have all been shown in other contexts to be extrinsic regulators of axonal regeneration. Thus, to address whether Dlk acts on RGC axonal regeneration via a cell-intrinsic or a cell-extrinsic mechanism, we transplanted small groups of retinal progenitor cells from the eye anlagen of *dlk* KO embryos into those of WT embryos. Transplantations were done at a stage prior to RGC genesis, but donor cells carried two co-integrated transgenes that would be expressed selectively in the descendent RGCs: a membrane-localized GFP and a membrane-localized mCherry (Isl2b:mem-GFP/mem-mCherry). The hosts carried a single transgene (Isl2b:GFP) such that their RGC axons expressed only cytoplasmic GFP. We subjected these animals to ONC at 8 dpf and live imaged the crushed optic nerves using a spinning disk confocal microscope with single axon resolution at 1, 3, and 7 days post-ONC. At 1 day post-ONC, GFP fluorescence was largely confined to the nerve region proximal to the injury site, with much weaker GFP fluorescence distal to the injury (Fig. S2A). Given the lower proportion of donor *dlk* KO axons in the nerve, most of this signal derives from the WT host axons. The donor *dlk* KO axons, uniquely labeled by the mem-mCherry reporter, were also largely confined proximally to the injury site, with somewhat more residual fluorescence distally, consistent with the predicted slower loss of fluorescence of mCherry relative to GFP. The distal tips of *dlk* KO axons displayed prominent enlargements, morphologically consistent with the retraction bulbs typically observed after axonal injuries (35, 36). These data suggest that Dlk absence has little if any effect on Wallerian degeneration or the initial morphological response to axon injury. At 3 days post-ONC, the retraction bulbs of the mCherry-labeled *dlk* KO axons were less prominent and showed little to no regrowth past the injury site, especially as compared with the GFP fluorescence derived from the WT axons, which was markedly higher distal to the ONC site than at 1 day post-ONC (Fig. S2B). By 7 days post-ONC, GFP fluorescence was uniform across the nerve, indicating extensive regeneration of the host WT axons. However, the mCherry-labeled *dlk* KO axons showed little regrowth, and the very small number of mCherry-labeled axons that did grow past the site of injury could have been newly born CMZ-derived RGCs, as CMZ also derives from RGC progenitors. While these experiments do not exclude the possibility that Dlk might be acting in either the progenitor cells or in other retinal cells derived from those progenitors, such as amacrine cells known to affect RGC regeneration (37, 38), they do rule out its action on the other surrounding cells in the optic nerve and suggest that the regenerative mechanism by which Dlk acts in *X. laevis* RGCs occurs within the injured RGCs themselves, as has been suggested in other contexts (34, 39, 40).

### Loss of Dlk does not affect either the innervation of the optic tectum during development or an optic tectum-dependent visually driven behavior but does affect the recovery of this behavior after ONC

**Fig. 5.**
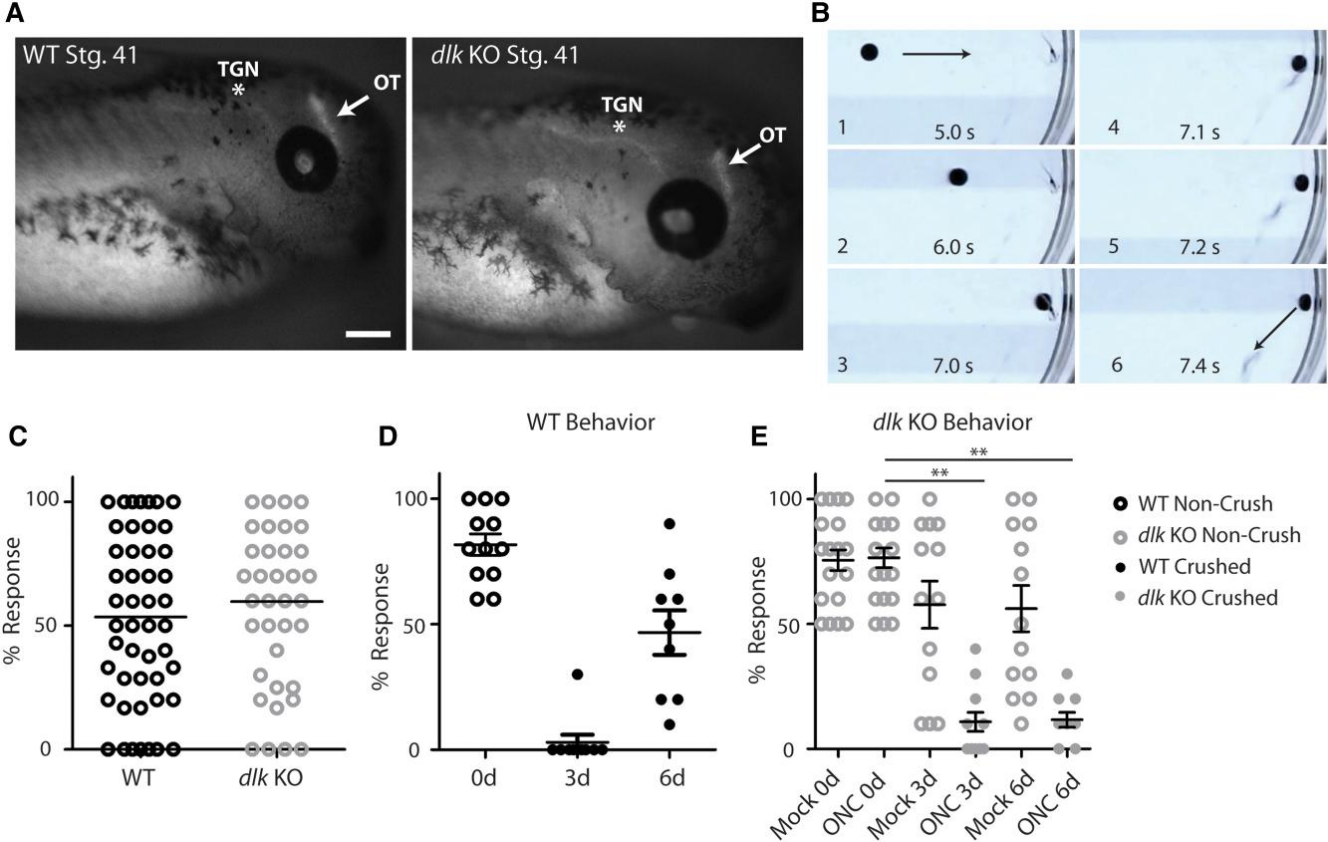
Absence of Dlk does not affect developmental optic tectum innervation by RGC axons or a visually guided behavior dependent on optic tectum innervation, but does block the restoration of the visually guided behavior after ONC. A) *dlk* KO animals show normal optic tectum innervation at NF stage 41. Both *dlk* KO and WT animals express an Isl2b:mem-GFP transgene. Note that Isl2b promoter expresses also in trigeminal neurons and sparse neurons in hindbrain and spinal cord, which also were not affected by KO of *dlk*. OT, optic tectum; TGN, trigeminal nucleus. Scale bar = 200 μm. B) A behavioral test of vision in *Xenopus laevis* tadpoles. A black dot stimulus projected from an LED screen beneath a glass-bottomed bowl is manually directed at the tadpole (still frames 1–3). If tadpole immediately darts away from the stimulus, trial is counted as a response (still frames 4–6). The percent response is then calculated after 10 mock collisions. C) Both WT and *dlk* KO animals show a similar range of responses to behavioral assays. Only animals that responded to 50% or more of the pretrials were included in subsequent ONC experiments. D) WT tadpoles subjected to bilateral ONC lose the dot-avoidance response by 3 days post-ONC, but largely regain it by 6 days post-ONC. E) *dlk* KO animals subjected to bilateral ONC but not mock-crush lose the dot-avoidance behavior 3 days post-ONC and do not recover it by 6 days post-ONC. “Non-Crush” *dlk* KO animals at 3 and 6 days were subjected to a mock crush following the prescreening prior to surgery.

To determine whether Dlk loss affects RGC axonal regeneration indirectly, either by generally affecting RGC axon outgrowth or by making the RGCs somehow dysfunctional, we assessed both the initial innervation of the optic tectum by RGCs during development and a behavior that depends on this innervation. We compared Isl2b:mem-GFP *dlk* KO embryos with WT embryos expressing the same transgene at Nieuwkoop and Faber (NF) stage 41, the earliest stage at which retinal projections have been documented to innervate the brain in *X. laevis* (41), and found that in terms of morphology and tectal innervation, *dlk* KO embryos were indistinguishable from WT embryos (Fig. 5A). To assess whether RGCs or the rest of the visual pathway were functionally affected by Dlk loss, we employed a collision-avoidance assay previously shown to assess functional vision in *X. laevis* tadpoles of this age (42) and which relies specifically on optic tectum innervation (43, 44). Tadpoles are placed in glass-bottomed bowls atop an LED screen on which a black-dot stimulus is displayed. The software user directs the black dot stimulus to move toward either a static tadpole or on a collision course with a slowly swimming tadpole. Ten trials of collisions (judged by the impending overlap of the stimulus with the tadpole head) are recorded and every trial in which the tadpole darts away from the black dot is graded as a response (Fig. 5B and Movie S1). First, to determine the effect of ONC on this behavior, we selected Isl2b:mem-GFP WT animals with over 50% response rates and then performed bilateral ONCs on half of those animals; such preselection of animals has been deemed necessary to exclude nonresponders (see (42, 44) and Supplementary Materials and methods). Crushed and naïve animals were then scrambled by one investigator, and the behavioral assay was repeated on the same animals at 3 days post-ONC by a second investigator unaware of which animals had received the surgery. This was immediately followed by live imaging to confirm complete bilateral crushes in the crushed cohort (defined as loss of all transgene fluorescence in both optic tecta). As expected, all tadpoles with complete tectal denervation had lost the ability to respond to the visual stimulus (Fig. 5D). However, by 6 days post-ONC, when the fluorescence intensity of the injured optic tecta had substantially returned, the majority of these animals were again able to respond to the dot stimulus. These results also suggest that ∼50% tectal innervation typically observed by 6–7 days post-ONC is sufficient to drive this visually guided behavior.

We then assessed the F1 progeny of *dlk* CRISPR/Cas9 F0s and found that the initial response was indistinguishable between WT and *dlk* KOs (Fig. 5C), demonstrating that Dlk does not affect the overall function of RGCs or the circuits needed for this visually guided behavior. Then, *dlk* KO animals with >50% response prior to ONC were divided into 2 cohorts, subjecting only 1 to bilateral ONC and reserving the other to ensure that *dlk* KO did not lead to a loss of avoidance behavior over the course of the experiment. *dlk* KO tadpoles also lost the ability to respond to the black-dot stimulus at 3 days post-ONC; but unlike the WT tadpoles, they did not recover this response at 6 days post-ONC (Fig. 5E). Taken together, these results demonstrate that Dlk does not affect either RGC axon outgrowth, the initial innervation of the optic tectum, or basic cellular processes within RGCs (at least those that convey functional vision); rather, Dlk acts specifically in axon regeneration and vision recovery after an axonal injury.

### *Dlk KO* specifically affects axon outgrowth from the injured RGCs

**Fig. 6.**
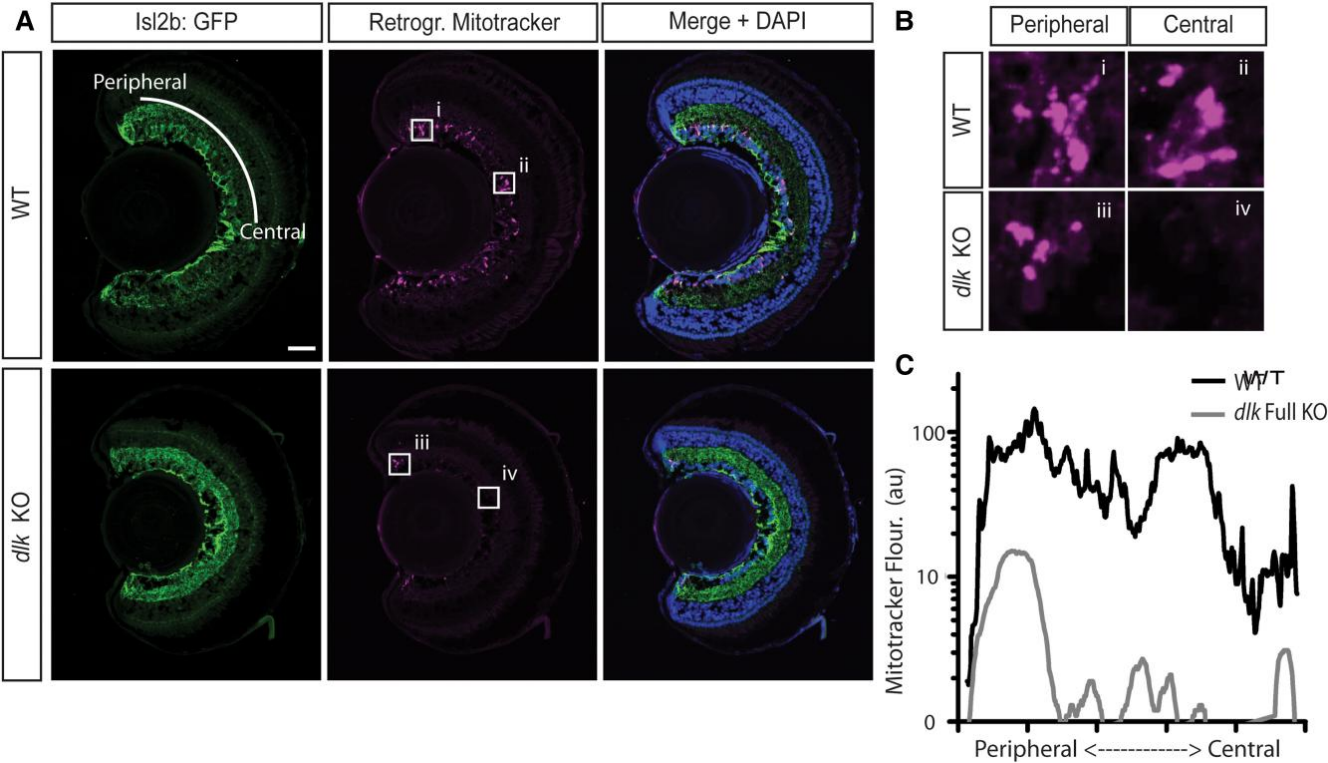
Dlk is necessary for optic tectum reinnervation by RGCs whose axons have been injured, but is dispensable for the optic tectum innervation by RGCs born at the CMZ after the injury. A and B) Tectal innervation assessed at 7 days post-ONC after implantation at 6 days post-ONC of Mitotracker into the optic tecta previously innervated by the crushed nerve. Retinal sections (A) and insets shown magnified (B) show that in WT animals, the tectal innervation 7 days post-ONC derives from RGCs throughout the retina, but in the *dlk* KO animals, it derives from only the peripheral-most RGCs, which are derived from the CMZ. Merges include nuclear labeling with DAPI. C) Average Mitotracker fluorescence in the ganglion cell layer as a function of location within the retina (from periphery to center). *N* = 10 *dlk* KO and 12 WT retinas. Scale bar = 50 μm.

Since the innervation of the optic tectum 6–7 days post-ONC derives from a mix of axons from the injured RGCs and the recently born CMZ-derived RGCs (see Fig. 2D and E), it is possible that the tectal innervation defects (see Fig. 4E) and nonrecovery of visual behavior (see Fig. 5E) observed in the *dlk* KO tadpoles could be due to the lack of Dlk in the new RGCs, the injured RGCs, or both. To address this question, we performed ONC on *dlk* KO and WT tadpoles at 8 dpf. At 6 days post-ONC, when live imaging confirmed that the injured optic tecta of WT tadpoles had reached approximately half the level of innervation of the uninjured control tecta, we performed retrograde tracing by insertion of a small Mitotracker-soaked fragment of Gelfoam into the injured optic tecta (Fig. 6). Immunohistochemical analyses of the retinal sections derived from these animals 1 day later found that in WT retinas, both the centrally located RGCs that were present prior to injury and the peripheral newly born RGCs were retrogradely labeled, demonstrating again that the tectal innervation 7 days post-ONC is a mix of regenerating and new RGC axons. However, in the *dlk* KO animals, the Mitotracker signal was limited almost exclusively to the peripheral RGCs, indicating that Dlk absence disproportionally affects the regeneration of the injured RGC axons. Notably, the fluorescence intensity of Mitotracker labeling in the periphery of the Dlk KO animals was lower than that in the WT animals. This observation could indicate that Dlk loss may affect all RGCs to some extent, directly or indirectly, or alternatively, their ability to be retrogradely labeled by Mitotracker, a possibility we directly tested (and excluded) below. Based on what has been observed in other systems, in the frog RGCs, Dlk could be acting locally in axons soon after injury or by leading to a transcriptional response once the axon injury signal reaches the nucleus; both possibilities were addressed next.

### Dlk does not affect changes in mitochondrial movement within RGC axons acutely induced by ONC injury

**Fig. 7.**
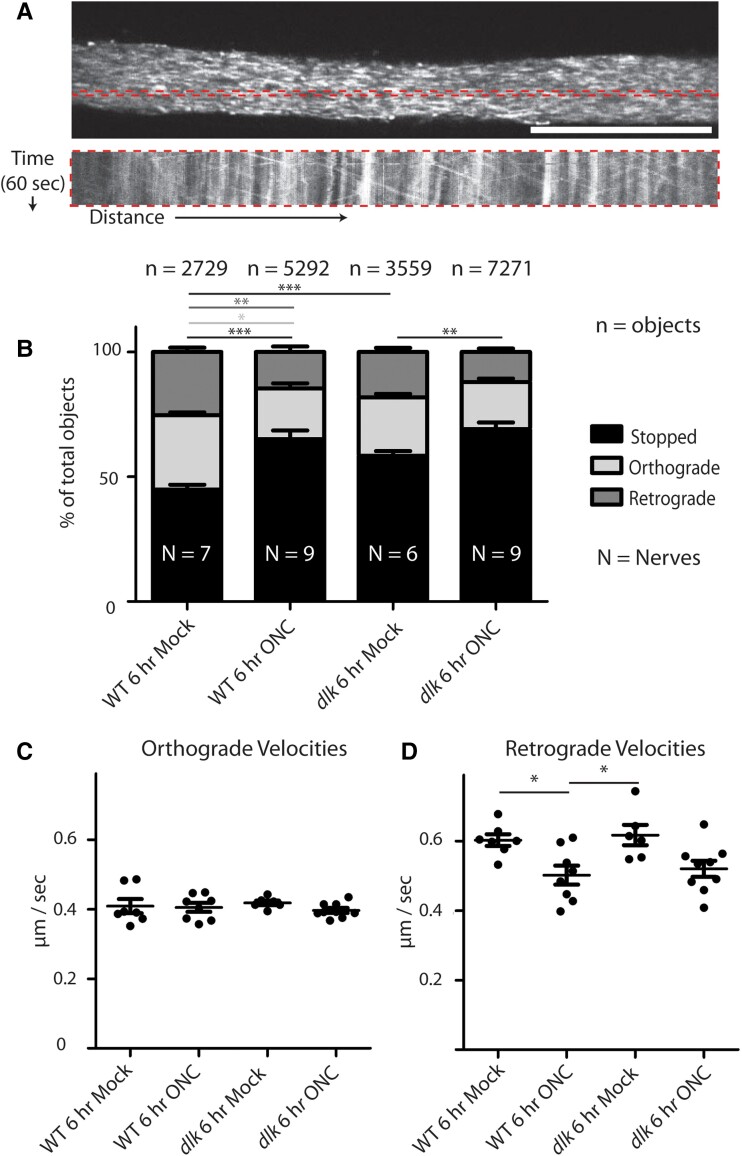
The absence of Dlk does not affect the ONC-induced change in mitochondrial movement behavior proximal to the crush site 6 h post-ONC. A) Single frame of 60, from 1 Hz 1 min live imaging of Mitotracker-labeled RGC axonal mitochondria. The dotted box indicates one of many regions of interest (ROI) per nerve analyzed through kymographs; the corresponding kymograph for that ROI shown below. Scale bar = 100 μm. B) In both WT and *dlk* KO nerves, ONC increases the percentage of stopped mitochondria at the expense of retrogradely moving mitochondria, relative to mock-crushed nerves. *N* = number of nerves per group; *n* = number of total mitochondria analyzed in all nerves per group. *P < 0.05, **P < 0.01, ***P < 0.001. C and D) ONC does, but *dlk* KO does not, affect retro-grade velocities 6 h post-ONC. C) Orthograde velocities not affected by either ONC or Dlk loss. D) ONC but not loss of Dlk decreases retrograde velocities. *P < 0.05.

In *Caenorhabditis elegans* motor neurons and murine spinal cord, Dlk has been proposed to act soon after the injury and locally within the axon itself by helping recruit mitochondria to the injury site (29, 34, 45). First, to test whether axon injury affects mitochondrial behavior in *X. laevis* tadpole RGCs, RGC mitochondria were labeled by intravitreal injection of Mitotracker 1 day prior to ONC, and the region of the optic nerve proximal to the injury site was imaged by spinning disk confocal microscopy 1 and 6 h post-ONC (Movie S2). In WT animals, ONC resulted in an increase in the number of immobile (stopped) mitochondria at the expense of mitochondria moving retrogradely along the nerve (from brain to soma) at both 1 and 6 h post-ONC (Figs. 7 and S3), consistent with previous studies of axonal injury (46, 47). ONC also resulted in a decrease in the velocity of retrogradely moving mitochondria at 6 h post-ONC. To test whether Dlk absence affected this transient change in the behavior of axonal mitochondria, *dlk* KO animals were similarly analyzed at 1 and 6 h post-ONC. The increase in stopped mitochondria at both time points post-ONC and the decrease in the velocity of retrograde movement at 6 h post-ONC occurred equally in the absence of Dlk (Figs. 7B and S3). While Dlk appeared to have a small effect on the velocity of retrograde movement after a mock ONC at 1 h post-ONC (Fig. S3), this effect was not observed at 6 h post-ONC (Fig. 7). Thus, while the tadpole ONC assay reliably causes changes in mitochondrial behavior after ONC, this effect is not altered in the absence of Dlk.

### Dlk KO eliminates the increase in phosphorylated Jun in RGC nuclei after ONC, but Jun does not appear to mediate the proregenerative action of Dlk

**Fig. 8.**
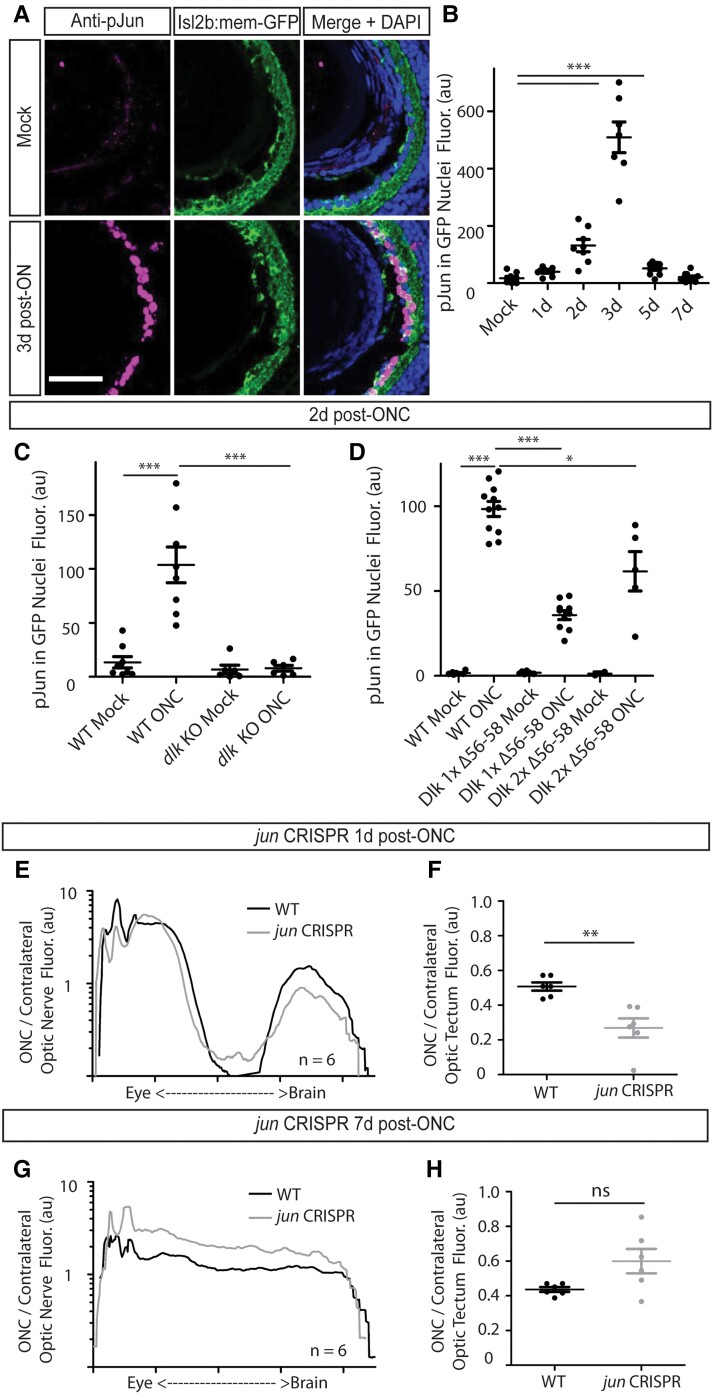
Dlk is essential for the large increase in phosphorylated Jun in RGC nuclei after ONC, but Jun is dispensable for regeneration. A and B) A large increase in phosphorylation and nuclear translocation of Jun within RGCs peaks at 3 days post-ONC. *N* = 7–11 retinas per time point, with a minimum of 3 cryosections averaged per retina. Merge includes nuclear labeling with DAPI. All significant values relative to Mock are shown. ***P < 0.001. C) *dlk* KO tadpoles display no nuclear pJun at 2 days post-ONC. *N* = 6 each for *dlk* KO Mock and ONC retinas, 9 for WT Mock retinas and 8 for WT ONC retinas, with a minimum of 3 cryosections averaged per retina. Scale bar = 50 μm. ***P < 0.001. D) Animals with either one or two copies of Dlk Δ56–58 show attenuated, but not absent, levels of pJun 2 days post-ONC. E) The distal crushed nerve in *jun* sgRNA–injected animals degenerates faster at 1 day post-ONC compared with WT animals. *N* = 6 nerves for each of the WT and *jun* CRISPR groups. F) The crushed optic tectum of *jun* sgRNA–injected animals becomes more extensively denervated at day 1 than that of uninjected WT animals. G) The relative (crushed/contralateral) optic nerve fluorescence in *jun* sgRNA–injected animals is higher across the length of the nerve than in uninjected animals at 7 days post-ONC. *N* = 6 nerves per group. H) The optic tectum of *jun* sgRNA–injected animals becomes reinnervated to an extent similar to that of WT controls, with a trend toward more regeneration.

The effects of Dlk on axonal degeneration and regeneration in other species have been shown to be largely mediated by a MAPK signaling cascade that ultimately leads to the phosphorylation and nuclear translocation of the transcription factor Jun, part of the AP1 complex, which then transcriptionally regulates many downstream genes (33, 34, 40, 48). To determine whether Jun is phosphorylated within RGCs following injury in our tadpole ONC model and if so, when, we performed ONC on WT tadpoles and examined retinal sections for the presence of phosphorylated Jun (pJun) in RGC nuclei at various time points post-ONC. We found a large increase in RGC nuclear pJun as early as 2 days, with the peak occurring at 3 days and returning to near-baseline levels by 5 days post-ONC (Fig. 8A and B).

Given that by 2 days post-ONC, pJun is still actively accumulating in the nuclei of injured RGCs but there is already a significant level of activation, we chose this time to test whether pJun in RGCs is dependent on the presence of Dlk. We find that in *dlk* KO tadpoles, there is essentially no RGC nuclear pJun at day 2 post-ONC (Figs. 8C and S4A), suggesting that Dlk is the sole upstream activator of Jun phosphorylation after ONC and, presumably, of many of Jun's downstream targets following axonal injury, despite *X. laevis* having an LZK homolog which in other systems can compensate for some of DLK's actions (31). Interestingly, partial KO of Dlk in animals that retained either 1 or 2 copies of the Dlk Δ56–58 deletion resulted in a reduction, but not a complete loss, of pJun signal (Figs. 8D and S4B), suggesting both that this small deletion results in a functional Dlk protein, and once again that Dlk acts in a dose-dependent manner.

In a previous study investigating the transcriptional response of *X. laevis* RGCs to injury, we found that Jun was upregulated transcriptionally more than 10-fold in frog RGCs at 3 and 7 days post-ONC in adult *Xenopus* (49). Based on this finding plus the necessity of Dlk for Jun phosphorylation in the current study, it seemed likely that RGC axonal regeneration might in large part be mediated via transcriptional changes downstream of Jun. To test this possibility, we selected the gene that is the clear homolog of the *jun* studied in mice, since all vertebrates have several *jun-*related genes (Fig. S5). We created F0 Jun KO animals in the background of the Isl2b:mem-GFP animals using an sgRNA that targets the first third of the Jun exon (Fig. S6). We subjected these sgRNA-injected animals to ONC at day 8 followed by imaging at 1, 3, and 8 days post-ONC. Animals were genotypically confirmed postexperiment to have >65% frameshift *jun* KO (Fig. S6B and C). Surprisingly, the crushed optic tectum of *jun* sgRNA–injected animals degenerated more completely by 1 day post-ONC than that of WT uninjected animals (Figs. 8F and S6E), and so did the nerve distal to the injury (Figs. 8E and S6D), demonstrating that the degree of KO in the F0 sgRNA–injected animals was sufficient to perturb Jun function. Furthermore, at 8 days post-ONC, at which time these control animals had obtained near 50% tectal reinnervation, *jun* sgRNA–injected animals did not have the decrease in reinnervation that would result if Dlk was regulating regeneration through Jun, but instead trended toward higher tectal reinnervation when compared with their uninjected siblings (Figs. 8H and S6G). The relative fluorescence across the crushed nerve compared with that of the contralateral nerve was likewise higher in *jun* sgRNA–injected animals (Figs. 8G and S6F). Thus, despite the large transcriptional upregulation of Jun after ONC (49) and the tight dependence on Dlk for Jun phosphorylation after axonal injury, activation of Jun itself does not appear to be necessary for RGC axonal regeneration.

## Discussion

In this study, we report the development of a novel RGC axon degeneration and regeneration assay in *X. laevis* tadpoles which enables relatively easy and inexpensive functional tests of genes in just 2 weeks, using in vivo imaging of axons within the optic nerve and tectum. Using this assay, we find that Dlk is essential for the regeneration of young *X. laevis* tadpole RGC axons after injury, and that it functions largely cell autonomously. We further find that Dlk is dispensable for the axonal outgrowth and tectal innervation of both early RGCs during development and new RGCs generated from the CMZ retinal progenitors following injury, and that in and of itself, it is dispensable for vision. Collectively, these findings position Dlk as an axon regeneration-specific gene. Finally, we find that phosphorylation of Jun post-ONC is completely absent in *dlk* KOs and dependent on Dlk dosage, but that, surprisingly, Jun itself does not seem to be necessary for RGC axonal regeneration.

Many distinct molecular pathways are activated in neurons after axonal injury (reviewed in Fague *et al*. (25)). In particular, DLK has been found to be a key mediator of cell death and regeneration in both central and peripheral nervous system (PNS) neurons (32). Following axonal injury, Dlk is phosphorylated at the injury site and then retrogradely transported back to the soma, where it activates the JNK1-3 pathways (33, 34). In *C. elegans*, *dlk* is required for regeneration in sensory motor neurons (29, 40); similarly, DLK initiates a proregenerative transcriptional response after sciatic nerve injury in mice (48). Additionally, the *Drosophila* homolog of *dlk*, Wallenda, functions in the injury-signaling cascade upstream of *jnk/fos*, and this signaling pathway is necessary for axonal regrowth following motor neuron injury (30). All these cases where there is a proregenerative action of Dlk involve invertebrates or mammalian PNS neurons, which, like the CNS neurons of *X. laevis*, possess an intrinsic capacity for regrowth. In the regeneration-deficient murine CNS, DLK activates both proapoptotic and proregenerative factors following injury by broad alterations of transcription, and loss of DLK eliminates the relatively modest RGC axonal regeneration induced by *PTEN* deletion (33). However, DLK has also been found to be necessary for RGC cell death in immunopanned primary murine RGCs, acting through the canonical JNK/MAPK signaling cascade. This makes DLK an attractive candidate for neuroprotection, including for RGCs; indeed, in cultured human stem-cell-derived RGCs, inhibition of DLK is highly neuroprotective (31). Our studies interpreted in the context of these previous studies support the view that in the CNS of all vertebrates, DLK plays an essential role in conveying a signal from the injury site in the axon back to the nucleus in order to trigger the appropriate transcriptional response. However, to our knowledge, this is the first demonstration of DLK being selectively necessary for regeneration (without affecting cell death) in a vertebrate CNS.

There are some limitations to our study that affect how much the results might be generalizable to adult mammalian RGCs. Our assays utilize a developmental system in which the damaged RGC axons regrow alongside axons from newly born RGCs, themselves largely impervious to Dlk loss. The axons from the noninjured RGCs might provide substrates, cues, or activities conducive to the growth of the regenerating axons; indeed, in our system, there is indirect evidence that they may pioneer the tectal reinnervation. That regeneration of the damaged axons occurs amid other uninjured axons in our system does not necessarily make it irrelevant to humans. In glaucoma, the most prevalent disease of RGCs where regenerative approaches might be implemented, the damaged axons do also largely co-exist with nondamaged axons; although, in glaucoma, these axons are fully differentiated rather than actively growing. Another limitation of the current study is that the tadpoles in which the nerves were crushed have axons that have not yet been myelinated, and myelin-derived proteins are potent inhibitors of regeneration (50). Both of these limitations would be addressed by assessing the function of Dlk and other genes in adult frogs as well, where the contribution of newly born RGCs at the ciliary marginal zone is very limited, and which through genetic manipulations could be completely eliminated. Finally, despite the significant genetic similarity of *X. laevis* to humans, other models that have been more extensively studied in the field of RGC regeneration are closer to humans evolutionarily than are frogs, raising the possibility that the relevant molecular machinery in frogs and humans may be somewhat different. Nonetheless, the fact that multiple species descended from the same vertebrate ancestor retain the ability to regenerate RGC axons suggests that mammals lost this ability over evolutionary time, so determining what is common and not between pro- and nonregenerative RGCs may be a productive path toward making axon regenerative therapies a real possibility.

Several other points raised by our own data also merit further consideration. The lack of effect of Dlk on mitochondrial movement after ONC stands in contrast to its reported effect in other species. While the mitochondrial effect of Dlk may have simply been missed by our assays, it could also be that in vertebrate RGCs, any effect of Dlk on mitochondria is irrelevant to whether a successful regenerative response is mounted. Perhaps of great interest, the dose dependence of the Dlk regenerative effect in frogs suggests the possibility that regenerative species may have more Dlk activity than species that do not regenerate, and that boosting Dlk activity might be therapeutically useful in mammals, perhaps even in humans. However, it will be critical to determine when and where Dlk action exerts this dose dependence, as MAPK pathways typically exhibit tight spatio-temporal control (51). Finally, the finding that the knock-down of *jun* does not phenocopy the RGC regeneration defect observed after *dlk* KO, but rather may accelerate the degeneration and regeneration of RGC axons, also merits further attention. It is possible that this is due to the incomplete loss of Jun (as assessed by quantification of frameshift mutations) achieved by our F0 CRISPR injections. However, the highly significant and consistent effect of our partial *jun* KO on RGC degeneration at 1 day post-ONC suggests that our level of *jun* KO was sufficient to produce a phenotype and that the effect (or lack thereof) on regeneration that we observed will hold when we analyze F1 animals in the future. In the PNS, Jun is upregulated in axons following injury (52–54) and is thought to promote axonal regeneration (55–57). However, these effects of *jun* may not involve the phosphorylation of its n-terminus by JNKs (58) and may, in fact, be due to its action in reprogramming Schwann cells to a regeneration-promoting state (59, 60); in all cases, the effect of Jun in PNS regeneration is rather limited. Thus, it is also possible that, because we used a global as opposed to conditional KO approach, the effects that we observed were due to the loss of *jun* cells other than RGCs, which could be tested in future by the retinal progenitor transplantation method used here for Dlk. Interestingly, our previous finding that a nearly 30-fold increase in Jun mRNA occurs at both 3 and 7 days post-ONC in adult frogs is similar to what occurs in mammalian PNS neurons (49). As such, it seems likely that another Jun-independent mechanism exists, which is even more important for regeneration in the vertebrate PNS, and our results here suggest that the same may be true for regeneration in *X. laevis* RGCs. As the genes downstream of Dlk are only partially known at present (49), comprehensive profiling studies could also be employed to map which genes are transcriptionally activated by Dlk in injured frog RGCs (in a dose-dependent manner and not dependent on Jun). Those genes could then be functionally tested by the CRISPR/Cas9 assays first described here.

Overall, our results demonstrate that Dlk is essential for successful RGC axonal regeneration in a species that maintains its CNS regenerative capacity. Our data support the view that it initiates a signaling cascade within the axon that is needed for nuclei to transcriptionally activate the intrinsic pathways required for axonal regeneration. We further believe that our current study reinforces the view that clinically relevant axonal regeneration may depend on a thorough deconstruction of evolutionarily conserved successful RGC axonal regeneration responses.

## Materials and methods

Details on materials and methods are available in Supplementary Materials and methods.

## Supplementary Material

pgad109_Supplementary_Data

## Data Availability

The authors affirm that all data necessary to evaluate the conclusions in this paper are included within the article and its supplementary materials. The Python cell-counting code previously published in Pittman *et al*. (13) is available at https://github.com/jbmiesfeld/Atoh7-remote-enhancer.
