# Supplementary material for: Dual leucine zipper kinase is necessary for retinal ganglion cell axonal regeneration in Xenopus laevis
Source: PNAS Nexus. 2023 Mar 30;2(5):pgad109. doi: 10.1093/pnasnexus/pgad109 (PMC10162689; doi:10.1093/pnasnexus/pgad109)
Supplement: pgad109_Supplementary_Data [file pgad109_supplementary_data.zip › PNASNEXUS-PNASNEXUS-2023-00079-T-s02.docx]

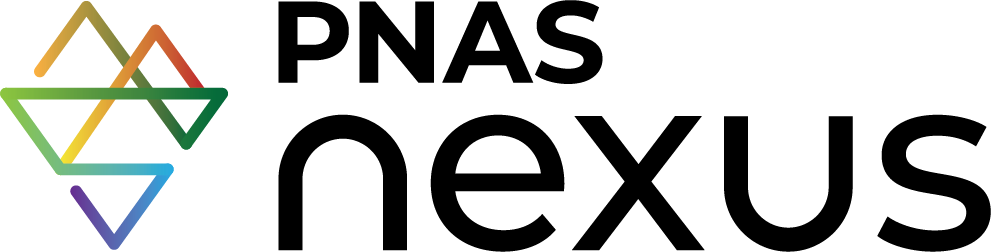


**Supporting Information for**

Dual leucine zipper kinase is necessary for retinal ganglion cell axonal regeneration in *Xenopus laevis*

**Authors:**

Lindsay Fague^1^, Nicholas Marsh-Armstrong^1^

^1^UC Davis Eye Center, Department of Ophthalmology & Vision Science, University of California, Davis, Sacramento, California, USA

**Correspondence:**

Nicholas Marsh-Armstrong
Department of Ophthalmology & Vision Science
University of California, Davis

1275 Med Science Drive Rm. 3451
Davis, CA 95616
Telephone: (530) 752-9420

Email: nmarsharmstrong@ucdavis.edu

**This PDF file includes:**

Supporting text

Figures S1 to S6

Legends for Movies S1 to S2

SI References

**Other supporting materials for this manuscript include the following:**

Movies S1 to S2

SI Materials and Methods

Animals

Wild-type and genetically modified *X. laevis* lines were housed in an investigator-maintained facility at UC Davis, and all work was carried out in accordance with protocols approved by the local IACUC. Tadpoles 2-wks and older were maintained in a drip-through *Xenopus* facility, and younger animals were maintained in 0.1X Modified Marc’s Ringer (MMR) in static glass containers.

Transgenic lines were created by restriction enzyme mediated integration (REMI) as first described in (1). Transgenic constructs and lines to express a cytoplasmic GFP in RGCs, *Tg(Isl2b:GFP)*, and a membrane localized mCherry in RGCs, *Tg(Isl2b:Mett7l-mCherry)* here referred to as Isl2b:mem-mCherry, have been previously described (2) and (3), respectively.

To be able to optimally label RGC axons with a membrane localized GFP, a four-step cloning strategy was used. First, an early version of GFP that fluoresces brightly in transgenic *Xenopus*, GFP3, where Kozak and ATG sequences were replaced by HindIII-EcoRV-NheI sequences, was cloned into the HindIII and XhoI sites of a pCS2 backbone using primers AAGGAGAAGCTTGATATCGCTAGCAGTAAAGGAGAAGAACTTTT and CGCTCGAG TTATTTGTATAGTTCATCCATGCC. Then, a 9 amino acid membrane localization sequence from lyn kinase was cloned into the HindIII and NheI sites using annealed kinased oligos AGCTTGCCACCATGGGATGTATAAAATCAAAAACAGACAATG and CTAGCATTGTCTGTTTTTGATTTTATACATCCCATGGTGGCA. This cDNA encoding lynk-GFP3 was then moved using the unique HindIII and XhoI sites into an intermediate that was then used to create the final construct pCS2(zf_Isl2b 20kb):lynk-GFP3 by recombineering, essentially as previously described (3); animals expressing this construct are here referred to as Isl2b:mem-GFP. Since the line used for most regeneration studies has a mem-GFP and mem-mCherry transgenes inserted into the same locus, these animals are here referred to as Isl2b:mem-GFP/mem-mCherry.

**Adult *Xenopus* ONC:** For adult frog ONC, the procedure was carried out as previously described (2, 5, 6). In brief, animals were anaesthetized in 0.5 g/L tricaine methanesulfonate solution (MS222). A small incision was made in the roof of the mouth using a sharp scalpel blade, followed by blunt dissection of the muscle and tissue layers using forceps to access the optic nerve, taking care to avoid injuring major blood vessels. The optic nerve was crushed for 4 seconds using #2 forceps. Animals were then allowed to recover in filtered 0.1x MMR solution for 1 day before being returned to the drip-through tanks until day of euthanasia.

**Young tadpole *X. laevis* ONC:** For tadpole ONC, two glass needles were pulled and then broken to 50-75 µm thickness, beveled to a 20-degree angle and mounted on micromanipulators. Tadpoles were anaesthetized in 0.2 g/L MS222 in filter sterilized 0.1x MMR solution and mounted on a custom stability plate (two glass rods affixed to the plate using modeling clay near parallel to one another; this shape supports the tadpole head on the rods with tail allowed to rest between the rods). Animals were positioned beneath a small fragment of Kimwipe dipped in the same anesthetic solution to prevent drying and to maintain adequate anesthesia for the duration of the surgery, with another small anesthetic-soaked fragment of Kimwipe placed just above the head across the glass stability rods to prevent drift. Surgery was performed using a Leica MZ10F fluorescent stereomicroscope to visualize the fluorescent ON. ONC was performed by inserting the two glass needles dorsally just adjacent to the ON and crushing by pressing the needles together. ONC were done monocularly except for animals to be used for behavioral assays of vision, in which case ONC was binocular. Tadpoles were allowed to recover in filtered 0.1x MMR + 20 mM HEPES + 50 µg/mL gentamicin pH 7.6 at 16 degrees for 12-15 hours. Following imaging 1d post-ONC, tadpoles were then kept at room temperature (RT) on a 12/12-hour light-dark cycle for the duration of the experiment. Any animals with either a full optic nerve transection (assessed visually at 1d and 3d post-ONC) or incomplete optic nerve crush (assessed by incomplete degeneration of the optic tectum at 3d post-ONC) were excluded from further imaging and analysis. In most experiments, tadpoles were kept in plastic boxes with dividers, with approximately 5 ml per cubicle to monitor the reinnervation response of every animal over time. In the case of the animals to be use in the behavioral assay, animals were housed together after ONC, as we find that schooling affects the robustness of the dot-avoidance response.

**Live imaging**: For lower resolution *in vivo* assessment of optic tecta and optic nerve denervation and reinnervation tadpoles were anaesthetized with 0.2 g/L MS-222 in 0.1x MMR and positioned in the same setup described above for tadpole ONC surgery. The same Leica MZ10F fluorescent stereomicroscope equipped with a PlanApo 1.0x lens (Leica, Wetzlar, Germany) that was used for the surgery was used again, this time along with custom iVision-Mac scripts (BioVision, Exton, PA) to capture images using a QImaging Retiga-Exi Monochrome Cooled 12-Bit camera (RET-EXI-F-M-12-C) (Teledyne Photometrics, Tuscon, AZ). These scripts prompted operators to focus and photograph each nerve and tecta individually using both GFP and mCherry filter sets. For the high-resolution imaging of RGC mitochondrial movement within axons and analyses of sparsely labeled axons, animals were also anesthetized with 0.2 g/L MS-222 in 0.1x MMR and then immobilized in 30 x 15 mm plastic dishes with custom Sylgard silicone molds previously created using gluteraldehyde fixed animals. These molds, with the addition of round 12 mm glass coverslips atop the animal and mold for stability, oriented the animals in such a way that the left nerve was parallel to the focal plane of the objective. Nerves were imaged using a Dragonfly spinning disc confocal microscope (Dragonfly 503 multimodal imaging system, Andor Technology, Belfast, UK) fitted with an 40x/1.1 (magnification/numerical aperture) HC PL APO water immersion objective and a Leica DMi8 inverted microscope (Leica, Wetzlar, Germany). Images were captured using an iXon Ultra 888 EMCCD camera and Fusion Software (both from Andor Technology, Belfast, UK) and laser lines of 50 mW 488 nm, 50 mW 561 nm, and 100 mW 643 nm. T-stack images for each animal recorded for 1 min at 1 Hz. Z-stack images scanned the entire length of visible nerves in 3-4 overlapping sections, scanning the entire thickness of the ON at each location at 1 µm steps, and using 2-frame averaging to increase resolution.

**Slide imaging:** For whole retina flatmounts (Figure 2) and retina sections (Figures 2, 6, and S4B), images were captured using the same Dragonfly spinning disc confocal microscope (Dragonfly 503 multimodal imaging system, Andor Technology, Belfast, UK) except using a 20x/1.1 (magnification/numerical aperture) HC PL APO objective For Figure 8A and Figure S4A retina sections images were captured using an Olympus FV3000 laser-scanning confocal microscope (Fluoview FV3000, Olympus Life Sciences, Waltham, MA) fitted with a 20x/0.7 (magnification/numerical aperture) UPLanSAPO objective. Images were captured using an Olympus FV31-LETD camera and Fluoview FV31S-SW Software and laser lines of with 405 nm, 488 nm, 561 nm, and 730 nm on GaAs detectors.

**Retrograde tracing:** For retrograde tracing, small Gelfoam® (Pfizer) strands visible only under the dissection microscope were soaked with 1 µL of Mitotracker DeepRed FM (Cell Signaling #8778S) at a concentration of 50 µg/µl until all Mitotracker solution was absorbed. Once dried, the Gelfoam® strands were further broken into smaller pieces of 5-15 µm diameter using forceps. Anesthetized tadpoles were placed in triangular notches carved into poured paraffin, removing most solution to anchor the animals sufficiently for the surgery. A small piece of Kimwipe soaked in anesthetic was used to cover tadpole to prevent drying and maintain adequate level of anesthesia. A pulled solid glass rod (Harvard Apparatus GR100-15) broken blunt and mounted on a micromanipulator was used to pierce the skin overlaying the rostral half of the optic tectum contralateral to the ONC. Using a 00 insect pin, the Mitotracker soaked Gelfoam fragments were placed overtop the created holes, followed by their insertion into the tectal neuropils with the aid of the solid glass rods. After surgeries, tadpoles were allowed to recover in filter sterilized 0.1X MMR at 16 ^o^C for 12 hr and then kept at RT for 6 hr prior to euthanasia and tissue collection for cryosectioning.

**Axonal mitochondria labeling and live imaging:** For live-imaging of mitochondria movement within RGC axons in the optic nerve, Deep-Red Mitotracker at a concentration of 200 µM diluted in filter sterilized 0.5x MMR was injected intravitreally using a Narishige IM 300 Microinjector fitted with glass needles pulled and cut to approximately 1 µm diameter. 40-80 nL of solution was injected per eye, as calibrated by comparing droplet sizes injected into mineral oil compared to those injected using a Drumond Nanodrop II piezo-controlled injector. Successful injections were verified by observing a small swelling of the eye. Animals were allowed to recover in filter sterilized 0.1X MMR for 24 hours at RT prior to ONC. Live imaging was carried out 1 and 6 hr post-ONC using the same Leica Andor dragonfly confocal microscope with 40x/1.1 N.A. water immersion objective as described above.

**sgRNA design and synthesis:** sgRNA design and CRISPR KO creation was based on protocols previously described for *X. laevis* (7, 8) with the following modifications. For sgRNA design, short sequence regions of *dlk* with 100% conservation between S and L chromosome were selected for input into CRISPR sgRNA design machine at <https://chopchop.cbu.uib.no/> and candidate sgRNAs to test were selected based on low number of predicted potential off target sequences (0 targets predicted to have 1 mismatch, and maximum of 1-3 off-targets predicted to have 2-3 bp of mismatch). sgRNA consensus regions in which all mismatches occurred within the 5 base pairs at the 5’ most end were excluded, as this has been suggested to decrease specificity and increase off target cuts (9). sgRNAs were ordered and synthesized as described in (7) except at the last step, sgRNAs were diluted to 800 ng/uL and snap-frozen on dry ice in single-use aliquots for injection. Final *dlk* sgRNA consensus sequence region chosen for this study was 5’-GACAGACCATGTCGGGCATTGGG-3’. Determination of which Jun to KO in frog was based on a multiple protein sequence alignments of Jun-related genes using ClustalW-Omega. Final *jun* sgRNA consensus sequence region chosen for this study was 5’- CAAGCTCGCTTCCCCGGAGTTGG-3’.

**CRISPR microinjections:** For sgRNA injection, *X. laevis* eggs were fertilized by overlaying them with macerated testes for 5 minutes prior followed by immediate dejellying with 25 g/L cysteine at pH 8. Eggs were quickly immobilized in custom mesh-inlaid 30 x 14 mM dishes in 0.1X MMR + 0.4% Ficoll + 20 mM HEPES + 50 µg/mL gentamicin pH 7.6. 0.5 µL of Cas9-NLS protein at 6.3 mg/mL (Macrolab; UC Berkeley) and 2.5 µL of 800 ng/µL sgRNA were mixed together and 20-40 nL of this mix injected into each embryo through 1-2µm tip diameter pulled glass needles controlled by a Narishige IM 300 Microinjector, with injection volume calibrated as above. Injected embryos were then moved into 0.1X MMR + 0.4% Ficoll + 20 mM HEPES + 50 µg/mL gentamycin pH 7.6 and placed at 16 ^o^C overnight to recover. Injections were done in small batches so that all were complete within 30 minutes of fertilization.

***Xenopus* genotyping:** For genotyping F0 sgRNA-injected embryos, 5 embryos were pooled together at NF stages 10-11.5 (approx. 24 hpf). For genotyping of F0 or F1 tadpoles, animals were euthanized by MS-222 overdose and 0.25-0.4 cm of tail tissue collected separately for each embryo. For genotyping of frogs, animals were fully anesthetized and a 2 mm fragment of webbing tissue was taken from one hindlimb using an Integra Miltex 2 mm biopsy punch (8-MIL-33-31-EA). Following tissue collection, tissues were snap-frozen on dry ice if genomic DNA extraction could not proceed on the day of tissue collection. In all cases, genomic DNA was prepared from tissue samples using Qiagen DNEasy kit. For genotyping of *dlk* F0 and F1 animals, a ~600 bp region was then PCR amplified around the predicted PAM site using the following primers: L chromosome, 5’-CTTGATGAGTACCTGGTAATACTCTTGGTAGG-3’ and 5’-GCTAATGACTTCAGCTTGGGATTTAGGGAAGG-3’; S chromosome, 5’-CCTTGATGCCTGGTAATACTTGTGGTACTTTC-3’ and 5’-GGCCTCAGAAGCTGCTATTAGGATTTTAGCT-3’. PCR products were then sequenced using primers 5’-CTTGATGAGTACCTGGTAATACTCTTGGTAGG-3’ for L chromosome and 5’-GGTACTTTCCTTGACTTGCAC-3’ for S chromosome. For genotyping of *jun* sgRNA injected animals, a ~1000 bp (for L) and ~800 bp (for S) region was PCR amplified around the predicted PAM site using the following primers: L chromosome, 5’- TTGTGGCGTCCGGACTGCGAGGAGCTCTCTGCTGC-3’ and 5’- CCAACCTGGAAATCCTCTCCAGCTTCCTCTTTCTG-3’; S chromosome, 5’- CTCCCACAATGCTCGGCAGGATACTGACAGTTGTA-3’ and 5’- CCTGGAGCCTGGGGTGCTGCACTGGCATTGGTGGG-3’. Sequencing returned .ab1 files which were then analyzed by TIDE (Tracking of Indels by Decomposition) analysis (10) via comparison to an unmixed control (no indel) sample from uninjected WT embryos amplified using the same primer sets.

**Behavior assay:** Software used to drive the tadpole visual avoidance assay was as previously described in (11). Dish radius was set at 210-235 px, background set to 160, travel time at 2500 px/sec and dot radius at 15 px. Monitor used for projection of the visual stimuli onto the tadpoles was a LiteMax 10.4” LED Backlight Monitor with brightness of 800 cd/m^2^ monitor (model SLD1055-ENA-B01-02, 10.4” TFT LCD, LED Backlight, 800nits, XGA). Recording of each trial was done using a Logitech HD Pro Webcam C920, 1080p Camera. Prior to behavioral experiments, all tadpoles were kept in groups so that animals could school and were maintained on a 12/12 light-dark cycle with daily feeding. For the behavioral assay, an individual tadpole was transferred into a 60 mm straight-sided glass dish with approximately 1.5 cm of 0.1X MMR depth placed atop the monitor on which software projecting the visual stimulation was displayed. Room lights were darkened during trials to ensure maximum contrast between the backlit monitor and the visual dot stimuli displayed. For each trail, user manually tracked tadpole movement and directed movement of a black dot stimuli onto the stationary tadpole or onto the path of a slowly swimming tadpoles. A trial was recorded as response if tadpole immediately changed speed/direction and moved away from dot upon overlap; if the velocity or direction of tadpole travel showed no change upon dot collision the trail was recorded as no response. Ambiguous trials were not counted. Ten informative trials were performed for each tadpole with a minimum time of 20 seconds between each trial. In the case of comparing WT to *dlk* KO animals in the absence of any insult, the behavior of all animals assayed was counted. For animals that were to be used in an ONC experiment, an initial screening was carried out prior to ONC. Tadpoles with a 50% or higher rate of response were retained for later experimentation, to exclude possible non-responders. Following such selection, tadpoles were subjected to bilateral ONC and allowed to recover in 0.1x MMR + 50 µg/mL gentamicin + 20 mM HEPES overnight at 16 ^o^C, then moved back into 12/12 light-dark cycle at RT. At 3d post-ONC, tadpoles were examined for complete ONC and any tadpoles with incomplete tectal denervation were excluded. Tadpoles with complete bilateral ONC were re-assayed using visual dot avoidance behavioral assay and moved into singly-housed 15 x 30 mm dishes for individual tracking. Tadpoles were maintained singly housed on 12/12 light-dark cycle and fed daily until final behavioral assay was performed again at 6d post-ONC.

***Ex vivo* analyses.** For adult tissue dissections, animals were euthanized by MS-222 overdose followed by decapitation. Brain was removed and flat-mounted by a partial-width incision along the tectal midline to open out both optic tectum lobes for imaging. Tecta were immediately imaged upon dissection. For tadpole tissue dissections, animals were euthanized by MS-222 overdose and fixed in 4% PFA for 4 hr at 4 ^o^C for cryosectioning or 1 hr at 4 ^o^C for retinal flatmounts. After fixation, eyes were removed from brain using 00 insect pins and placed in sucrose overnight at 4 ^o^C (for cryosectioning) or in methanol overnight or longer at -20 ^o^C (for flatmounts). For tadpole retinal flatmounts, the lens was removed and eye flattened using 4-8 scalpel blade cuts placed symmetrically around the eye perimeter, then pressed open between a coverslip and glass slide in Aquamount mounting media. For cryosectioning, eyes were embedded in OCT, frozen in dry ice, and then sectioned in 10 µm sections onto gelatin-coated slides. Slides were blocked for 30 min in 10% normal goat serum (NGS) in phosphate buffered serum with 0.1% BSA and 0.1% TritonX-100 (PBT), then incubated ON at 4 ^o^C in primary antibodies diluted in PBT with 5%NGS, washed in PBT, followed by 4 hrs at RT in secondaries in PBT, additional PBT washes and counterstaining with Dapi. Primary antibodies included: Aves GFP #GFP-1020 (1:500), BD Pharmingen anti-BrdU #555627 (1:500), Clontech Living Colors mCherry #632543 (1:500), Cell Signaling Technologies P-c-Jun #9261S (1:200), and Abcam mCherry #ab167453 (1:500). Secondary antibodies included: Jackson ImmunoResearch Cy3 anti-mouse IgG #115-165-146, Jackson ImmunoResearch AlexaFluor 488 anti-chicken #103-547-008, Jackson ImmunoResearch AlexaFluor 647 anti-rabbit #111-605-144, Jackson ImmunoResearch Cy5 anti-mouse #115-175-146, and Jackson Immunoresearch Cy3 anti-rabbit IgG #111-165-144. All secondary antibodies were used at 1:250 and passed through a 0.2µm filter before use. For birthdating experiments with BrdU, 250 nL BrdU was injected into tadpoles intraperitonally at a concentration of 20 mM made up in 0.5x MMR. For birthdating experiments with EdU, tadpoles were immersed (non-anaesthetized, free-swimming) in a solution of 0.1X MMR + 10 mM EdU for 20 minutes.

**Script to quantify optic nerve and optic tecta axon degeneration and regeneration:** A custom IPlab (Biovision, Exton, PA) script was created in which both tecta (crushed and contralateral) are individually traced and then their fluorescence normalized by a background subtraction step using an area of the tadpole head immediately adjacent to the tecta that lacked any melanocytes. Each tecta’s fluorescence values 10% above background were subjected to a subsequent autosegmentation step which excludes all pixel values within the traced region which fall below 10 % below background and thus likely represent melanocytes within the traced regions. Following this normalization, both area and mean fluorescence measurements were taken for both tecta, and the values for crushed tecta divided by the values for the contralateral tecta to return a measure of relative fluorescence of crushed tecta compared to the fluorescence of control. Lines were then manually drawn over crushed and contralateral nerves and then thickened to about two nerve diameters, normalized in length to an arbitrary value of 600, and then used to extract the fluorescence intensity values across the central 400; values at either end were excluded to account for the out-of-focus regions nearest the head and the eye which are unavoidable in live-imaging the *X. laevis* optic nerve at this stage. This was then followed by a two-step normalization. First, background subtractions are done individually for each nerve using a background location selected by user adjacent to each nerve and free of melanocytes. Next, every value on either crushed or contralateral nerve is set to a minimum value of 10 to account for any values of 0 which might have been returned from the background subtraction due to melanocytes in the ROI; this avoids division by 0 and infinitely high values in following step. Finally, the values along each of the 400 points along the nerve were divided by the value of the contralateral nerve at the equivalent location. In the case of the sparse labeling experiments, analyses were carried out on maximal intensity projections of composites aligned manually or through the Pairwise Stitching Fiji plugin. In order to compensate for large endbulbs or myeloid cells that produce sharp maxima that skewed analyses, small artifacts were manually excluded and compensated for, and histograms were smoothened by presenting all values as the average of the surrounding 50 points.

**Analyses of pJun and retrograde tracer in retina sections:** Custom IPlab scripts were used to analyze maximum intensity projections of 10 µm retina sections. For quantifying RGC nuclear pJun, three to six sections were analyzed per retina, and the RGC area was automatically segmented using the GFP color channel, followed by autosegmentation of Dapi, to isolate the RGC nuclei. Within these RGC nuclei, and in order to avoid introduce subjective choices that could bias results, the far-red (pJun) signal was then measured as the signal above 20 different stepwise thresholds, and while results were significant at most of these and changes in magnitude larger in different ones, the ones presented are the central threshold of those that attained above p<0.01 significance between any two groups, similar to what we have previously done (12). In the case of Mitotracker retrograde tracing, four to six sections were analyzed per retina. In this case the GFP and Dapi were not autosegmented but were used to manually draw a line through the entire ganglion cell layer. After automatically thickening the line to approximately 2 cell diameters, the Mitotracker associated AlexaFluour 647 signal was plotted as a function of distance from the retina periphery; to compensate for the very punctate nature of the Mitotracker signal, the histograms were smoothened by presenting all values as the average of the surrounding 50 points.

**Statistical analysis:** GraphPad Prism was used for graphing data and determine statistical significance for all experiments. Mean comparisons were tested for significance using a two-way ANOVA with Mann-Whitney post-test for all multi-group comparisons and a students’ T-test for all two-group comparisons. For all tests, a p-value less than 0.05 was considered significant.

**Supplemental Figures and Legends**

**
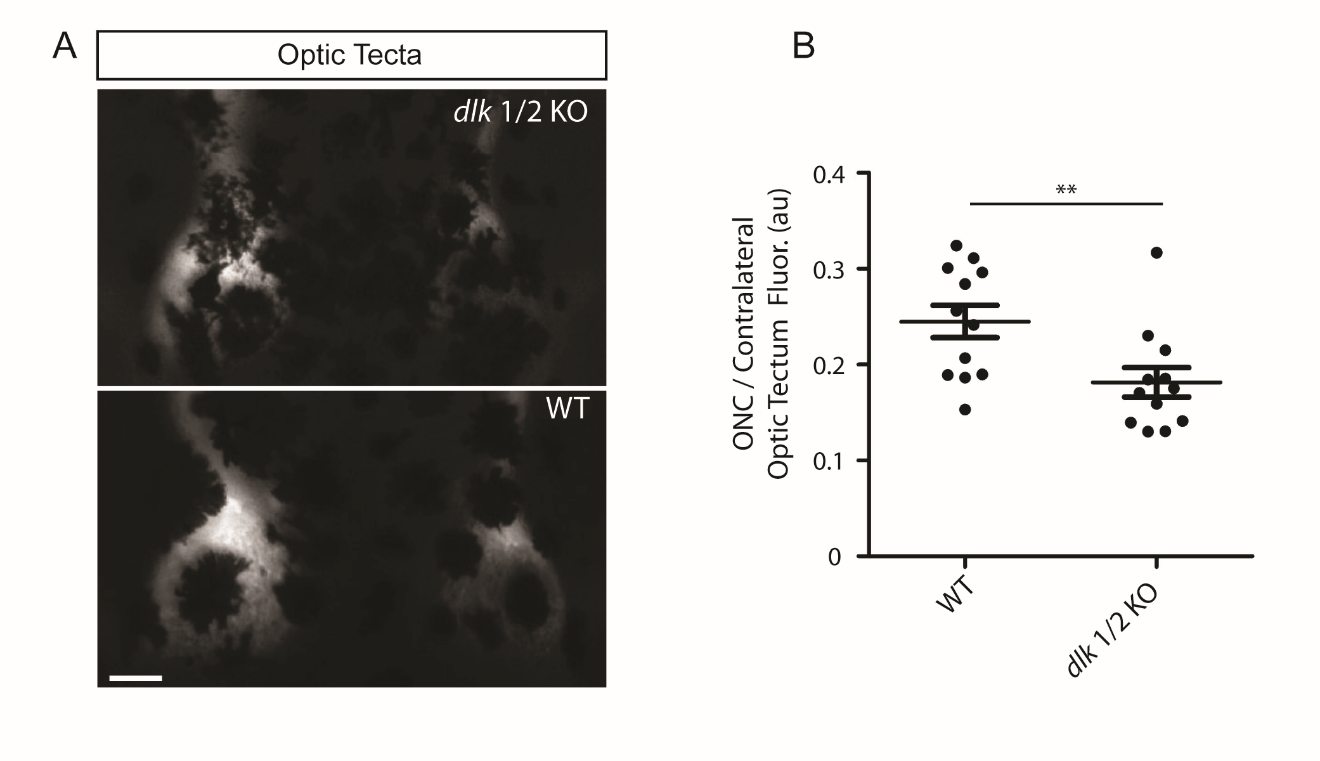
Supplemental Figure S1.** Animals with half the complement of Dlk shows a defect in tectal reinnervation 6d post-ONC. A. Representative images (closest to means). B. Measures of fluorescence. Scalebar = 100 µm. ***P* <0.01

**
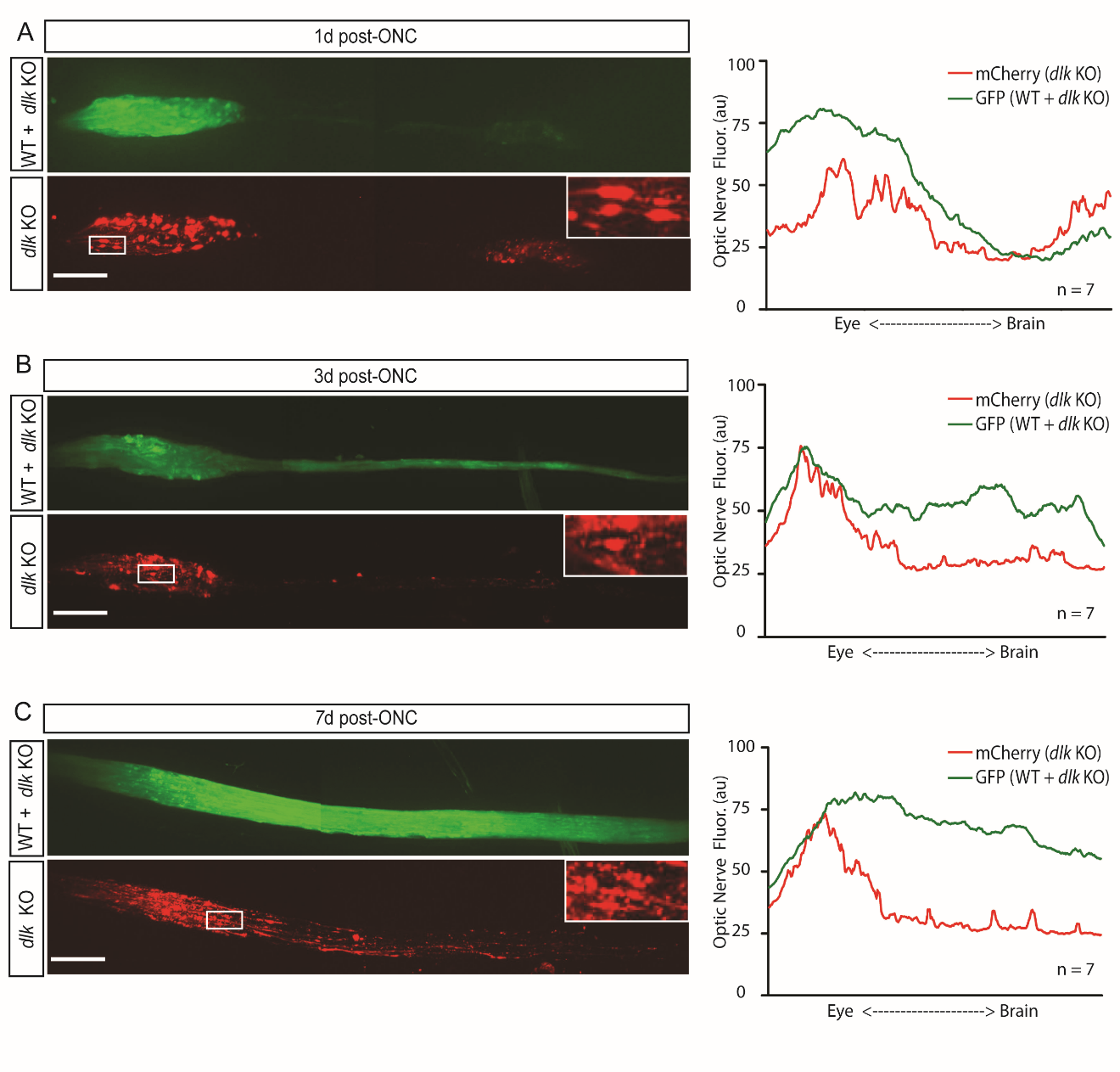
**

**Supplemental Figure S2.** Axons of *dlk* KO RGCs fail to regrow after crush in Wt optic nerve surrounded by regenerating WT RGC axons. A-C. Sparse *dlk* KO donor-derived RGC axons expressing membrane GFP and membrane mCherry reporters amid host RGC axons expressing a cytoplasmic GFP reporter. A. By 1d post-ONC, both donor and host axons have similarly degenerated past the crush site. Inset shows retraction bulb in mCherry-labeled *dlk* KO axons. B. By 3d post-ONC, the GFP-labeled WT axons but not the mCherry-labeled *dlk* KO axons have begun to grow past the crush site. C-D. By 6d post-ONC, even when large numbers of WT RGC axons have extensively regrown, most *dlk* KO axons remain proximal to the crush site. Note that some *dlk* KO axons do grow across the crush site; these likely represent axons from RGCs derived from CMZ progenitors. Scalebars = 50 μm.

**
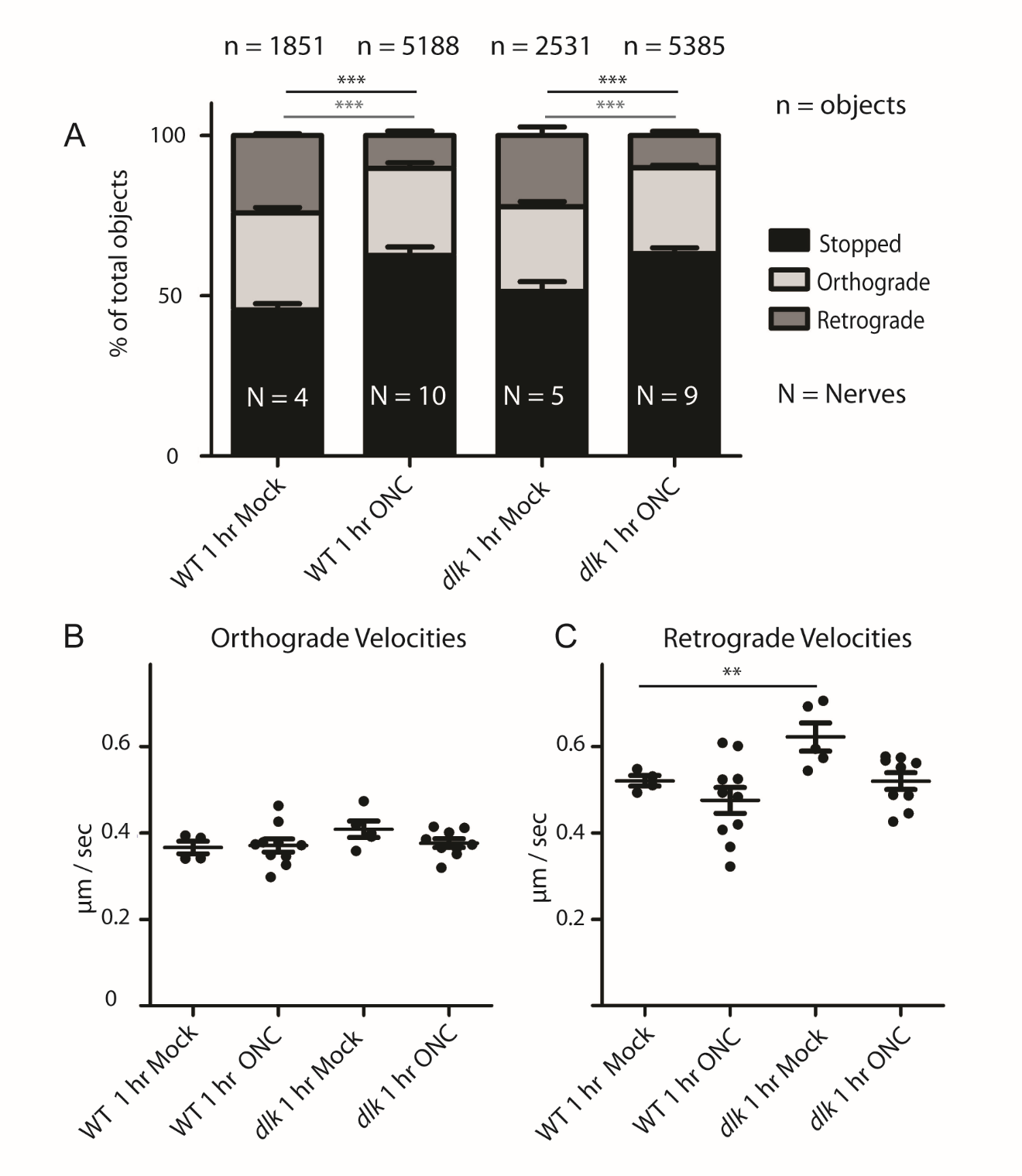
Supplemental Figure S3.** At 1 hr post-ONC, absence of Dlk does not affect the ONC-induced change in mitochondria movement proximal to the crush site. A. In both WT and *Dlk* KO nerves, ONC increases the percentage of stopped mitochondria at the expense of retrogradely-moving mitochondria. *Dlk* KO has no effect on the relative percentage of mitochondria moving in either direction. N = number of nerves per group; n = number of total mitochondria measured in all nerves per group. B. Neither injury nor *Dlk* KO affects orthograde velocities at 1 hr post-ONC. C. *Dlk* KO may have a small effect on retrograde velocities in Mock-crushed animals 1 hr post-ONC.

**
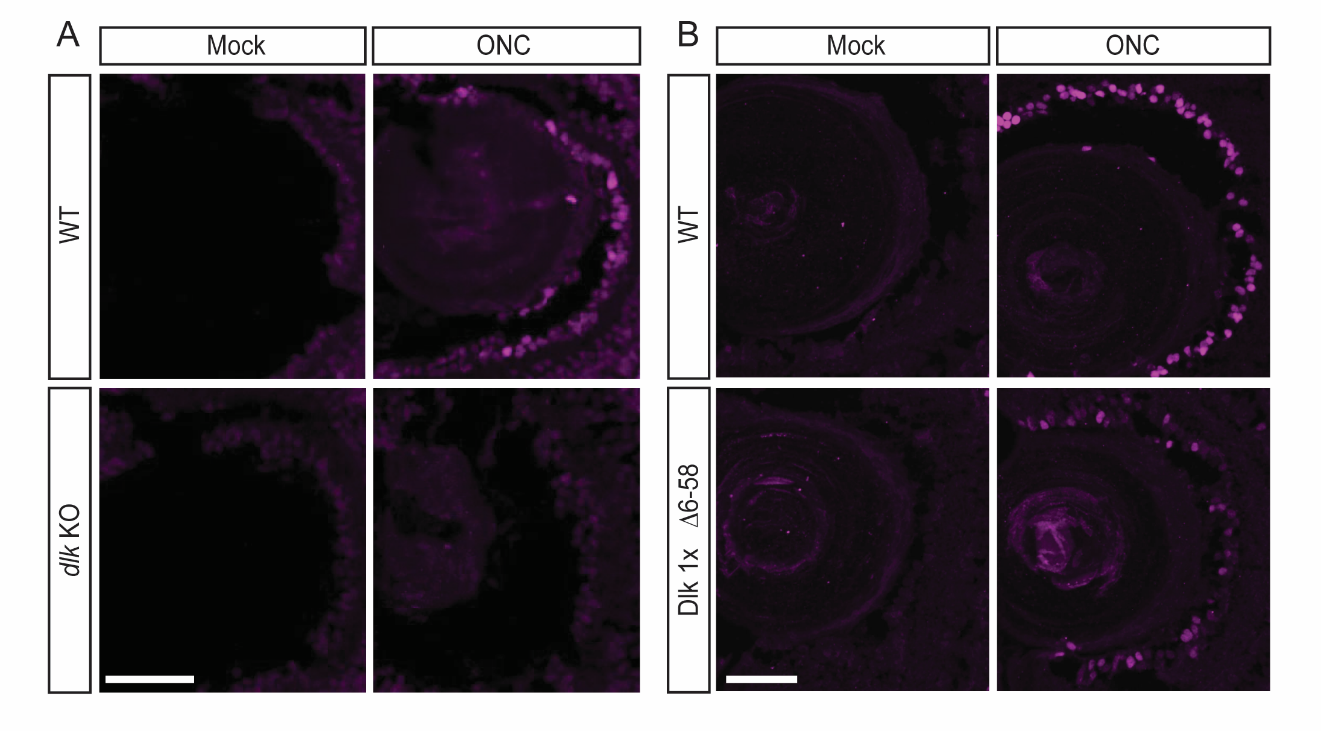
Supplemental Figure S4.** A. *dlk* full KO tadpoles display no nuclear pJun at 2d post-ONC. N = 6 each for *dlk* KO Mock and ONC retinas, 9 for WT Mock retinas and 8 for Wt ONC retinas, with a minimum of 3 cryosections averaged per retina. B. Animals with one copy of Dlk ∆56-58 show attenuated, but not absent, levels of phosphorylated Jun 2d post-ONC. Scalebars = 50 μm.

**
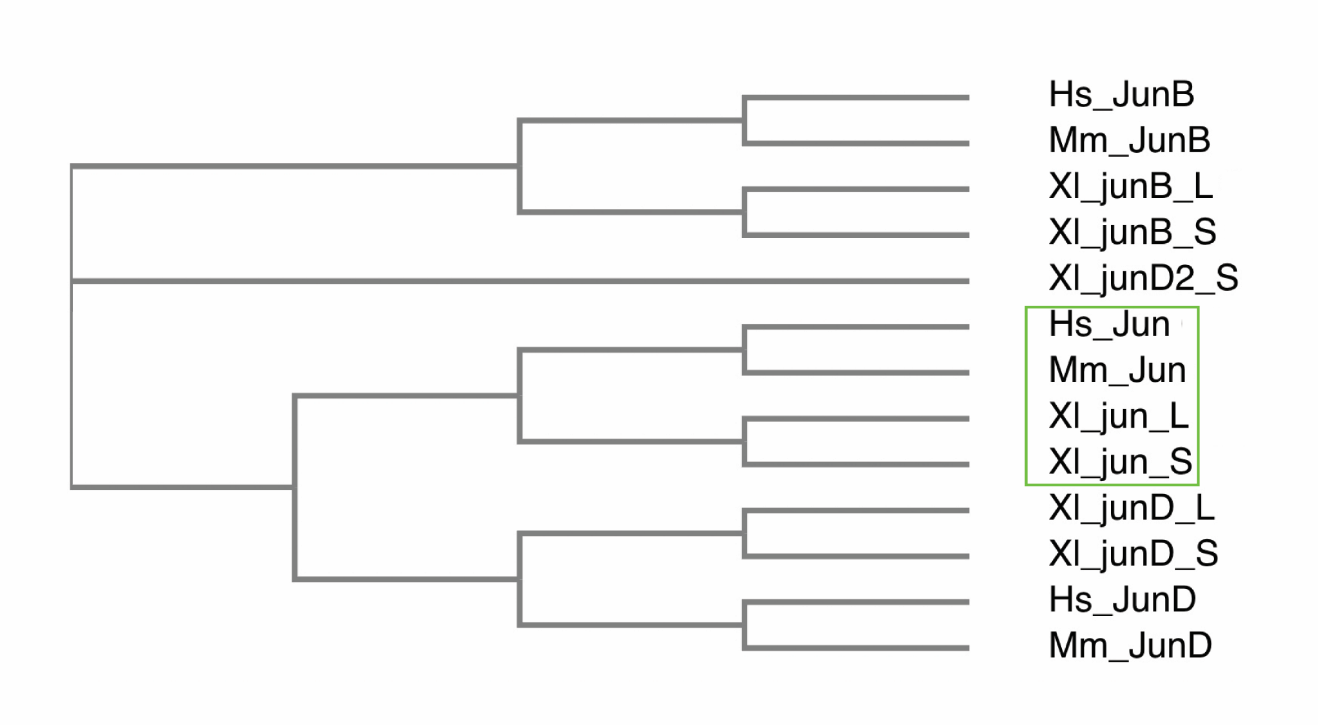
**

**Supplemental Figure S5.** Phylogenetic tree detailing the various *jun* homologs of *X. laevis* (Xl) compared to those of mice (Mm) and humans (Hs). Green box highlights that the two *jun* homologs targeted by our knockout model were the closest evolutionarily to mouse and human *jun*.

**
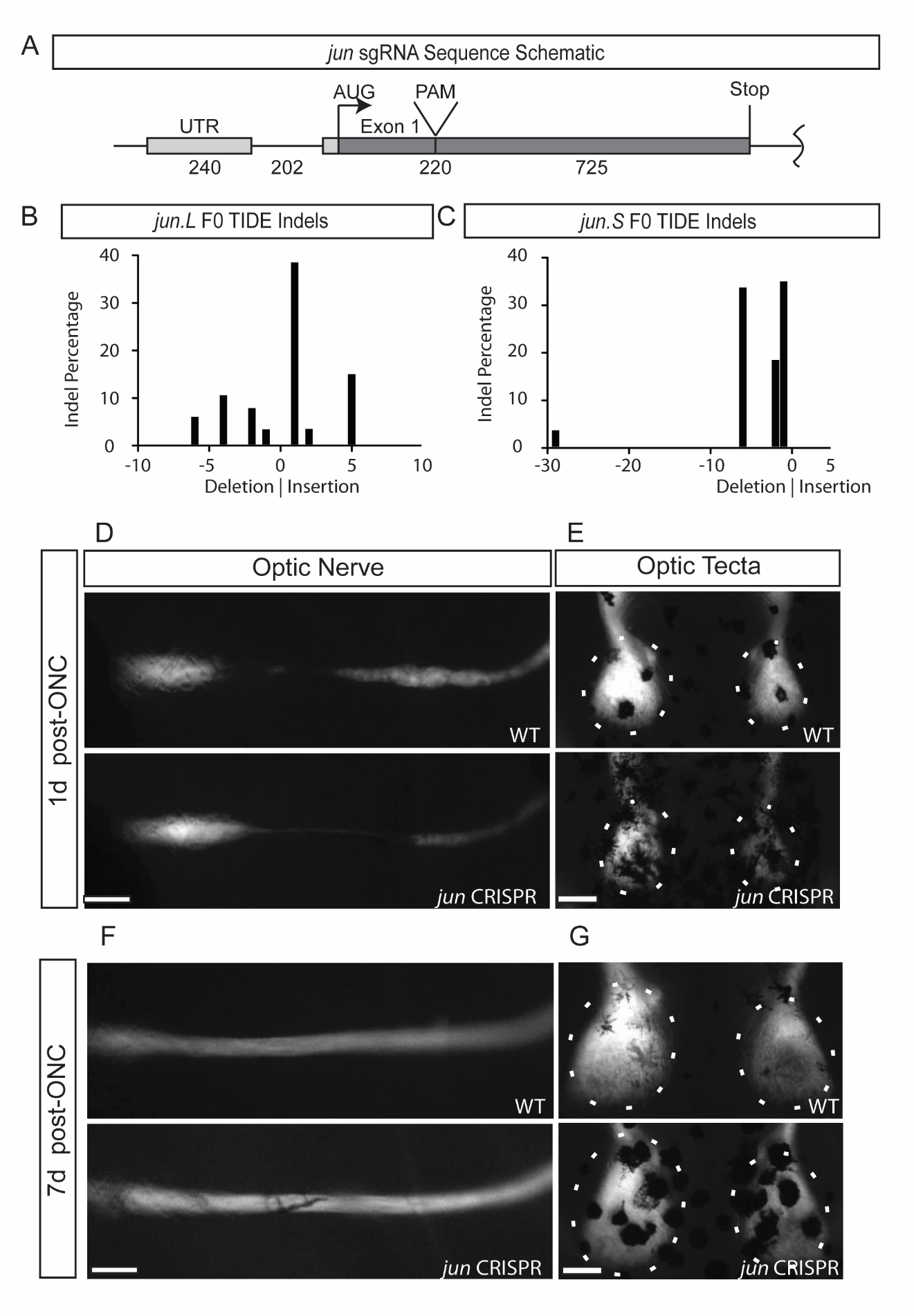
Supplemental Figure S6.** *jun* sgRNA injected animals only differ from uninjected animals in having accelerated degeneration. A. Schematic showing the location of the PAM for the sgRNA used for *jun*. B-C. *jun* gRNA F0 indel efficiency was >80% with over >65% frameshift mutations for both *jun.L* and *jun.S* genes. D. The distal-most portion of the crushed nerve in *jun* sgRNA-injected animals loses fluorescence moreso than that of WT animals at 1d post-ONC. N = 6 nerves for each of WT and *jun* CRISPR groups. E. The crushed optic tectum of *jun* sgRNA-injected animals also becomes denervated at d1 post-ONC. F. The fluorescence of the crushed nerve in *jun* sgRNA-injected animals appears somewhat higher than that of uninjected animals at 7d post-ONC. N = 6 nerves per group. G. The optic tectum of *jun* sgRNA-injected animals becomes reinnervated to a similar extent relative to WT controls.

**Movie S1 (separate file)**. Behavioral dot-avoidance assay. A single tadpole placed in a glass-bottomed bowl atop an LED screen allows for presentation of a black-dot stimulus. Upon user-directed movement of the stimulus such that it moves from the center to overlap with the tadpole, a tadpole which is capable of functional vision will change direction and velocity of swimming to dart away from the stimulus.

**Movie S2 (separate file).** Mitochondria live-imaged in the tadpole optic nerve. Imaging was captured at 1 Hz and here is displayed 7x speed (i.e., 7fps). Video is of a mock-crushed *Dlk* KO optic nerve at 1 hr post-surgery and 24 hours after intravitreal injection of Mitotracker dye. Note that approximately half of mitochondria appear stopped along the nerve, and there are relatively balanced numbers of mitochondria moving both retrogradely (towards the left) and orthogradely (towards the right).

**SI References**

1. Amaya E, Kroll KL. A method for generating transgenic frog embryos. Methods Mol Biol. 1999;97:393-414.

2. Whitworth GB, Misaghi BC, Rosenthal DM, Mills EA, Heinen DJ, Watson AH, et al. Translational profiling of retinal ganglion cell optic nerve regeneration in Xenopus laevis. Dev Biol. 2017;426(2):360-73.

3. Mills EA, Davis CH, Bushong EA, Boassa D, Kim KY, Ellisman MH, et al. Astrocytes phagocytose focal dystrophies from shortening myelin segments in the optic nerve of Xenopus laevis at metamorphosis. Proc Natl Acad Sci U S A. 2015;112(33):10509-14.

4. Das B, Brown DD. Controlling transgene expression to study Xenopus laevis metamorphosis. Proc Natl Acad Sci U S A. 2004;101(14):4839-42.

5. Belrose JL, Prasad A, Sammons MA, Gibbs KM, Szaro BG. Comparative gene expression profiling between optic nerve and spinal cord injury in Xenopus laevis reveals a core set of genes inherent in successful regeneration of vertebrate central nervous system axons. BMC Genomics. 2020;21(1):540.

6. Watson FL, Mills EA, Wang X, Guo C, Chen DF, Marsh-Armstrong N. Cell type-specific translational profiling in the Xenopus laevis retina. Dev Dyn. 2012;241(12):1960-72.

7. Bhattacharya D, Marfo CA, Li D, Lane M, Khokha MK. CRISPR/Cas9: An inexpensive, efficient loss of function tool to screen human disease genes in Xenopus. Dev Biol. 2015;408(2):196-204.

8. Willsey HR, Exner CRT, Xu Y, Everitt A, Sun N, Wang B, et al. Parallel in vivo analysis of large-effect autism genes implicates cortical neurogenesis and estrogen in risk and resilience. Neuron. 2021;109(8):1409.

9. Zhang XH, Tee LY, Wang XG, Huang QS, Yang SH. Off-target Effects in CRISPR/Cas9-mediated Genome Engineering. Mol Ther Nucleic Acids. 2015;4:e264.

10. Brinkman EK, Chen T, Amendola M, van Steensel B. Easy quantitative assessment of genome editing by sequence trace decomposition. Nucleic Acids Res. 2014;42(22):e168.

11. Khakhalin AS. Analysis of Visual Collision Avoidance in. Cold Spring Harb Protoc. 2021;2021(4).

12. Nguyen JV, Soto I, Kim KY, Bushong EA, Oglesby E, Valiente-Soriano FJ, et al. Myelination transition zone astrocytes are constitutively phagocytic and have synuclein dependent reactivity in glaucoma. Proc Natl Acad Sci U S A. 2011;108(3):1176-81.

13. Miesfeld JB, Ghiasvand NM, Marsh-Armstrong B, Marsh-Armstrong N, Miller EB, Zhang P, et al. The. Proc Natl Acad Sci U S A. 2020;117(35):21690-700.
