# Supplementary figures and images for: Dual leucine zipper kinase is necessary for retinal ganglion cell axonal regeneration in Xenopus laevis
Source: PNAS Nexus. 2023 Mar 30;2(5):pgad109. doi: 10.1093/pnasnexus/pgad109 (PMC10162689; doi:10.1093/pnasnexus/pgad109)

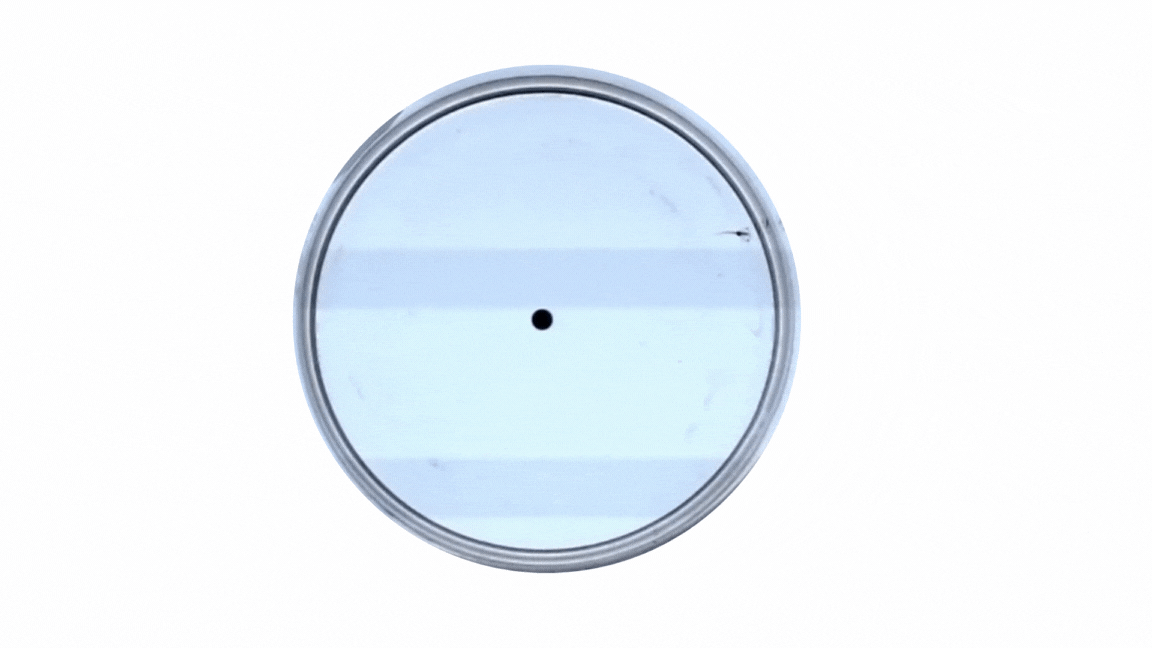

Supplement: pgad109_Supplementary_Data [file pgad109_supplementary_data.zip › PNASNEXUS-PNASNEXUS-2023-00079-T-s03.gif]
