# Supplementary material for: Dual leucine zipper kinase is necessary for retinal ganglion cell axonal regeneration in Xenopus laevis
Source: PNAS Nexus. 2023 Mar 30;2(5):pgad109. doi: 10.1093/pnasnexus/pgad109 (PMC10162689; doi:10.1093/pnasnexus/pgad109)
Supplement: pgad109_Supplementary_Data [file pgad109_supplementary_data.zip › PNASNEXUS-PNASNEXUS-2023-00079-T-s01.docx]

**January 25, 2023
Response to Reviewers**

We thank the reviewers for their insightful comments on the original version of our submitted manuscript. We are grateful for their kind review of the thoroughness and quality of our work and equally grateful for their input as to where we could expand our experiments and clarify or adjust our conclusions. We have attempted to address every point raised in the original reviews. We have moved one figure to supplementary to make room for the requested work on Jun, incorporated new paragraphs to specifically address reviewer comments, and have made additional small changes throughout the manuscript. We feel that our current manuscript is very significantly improved after having been so carefully reviewed, and have our best to make our revision as thoughtful as the feedback which we received.

A major point raised by both reviewers was that our emphasis on the importance of cJun in the *X. laevis* RGC axonal regeneration response might be overstated, especially as our initial conclusions relied solely on immunohistochemical analysis of pJun in the retina following ONC in Dlk KO frogs. We emphatically agree with his feedback and indeed, were already planning for additional experiments on cJun following submission of the original manuscript. The results, which we report here in this revised manuscript, involved CRISPR-based knockdown of cJun as suggested. The results validated the reviewers’ reservations and put to flight our initial assumptions regarding the importance of cJun to regeneration; much to our surprise, we find that c-Jun does not in fact appear to be required for RGC axonal regeneration in *X. laevis*, and indeed may even have an inhibitory action (Figures 8 and S6). We have also referenced (as per one review comment) the body of work that shows that c-Jun phosphorylation may also not be essential for axonal regeneration in the PNS. On that point, we now also reference our own previous work that shows Jun to be transcriptionally regulated after ONC more than tenfold in RGCs, similar its upregulation in the system in which that phosphorylation is non-essential for regeneration. Finally, we have included a new supplemental figure comparing the various *jun* variants (including the one we targeted) which are present in X. laevis and those present in mice and humans, showing this gene family to be highly (though not completely) conserved. This is a clear example where reviewer comments had a fundamental effect in changing the punch-line of a body of work. Thank you.

We also agree with the critique, voiced by both reviewers, regarding our description of our new method as a “screen” without elaborating on other targets than *dlk.* Indeed, the reviewer was correct in assuming that we might have screened dozens of other candidates but wished to reveal other hits independently. However, with only a single gene reported on in the initial manuscript our initial descriptor of this as “screen” was unwarranted. As such, we have modified our language here to better describe our method as an “assay”. We have also included details about a second gene (c-Jun) which we have assayed with this new method as this was a second putative regeneration mediator and, as mentioned above, to our surprise did not phenocopy the Dlk findings.

We also wholeheartedly agree with the reviewer that pointed out that differences exist between juvenile and adult members of any species. In response to this reviewer’s comment about these possible limitations of our model, we have added in a new limitations paragraph in our discussion a description of what we consider to be the two most salient and relevant differences between RGCs in tadpoles and adult frogs. However, we have also attempted to make more explicit what we consider to be the principal advantage of our system relative to other available systems to query RGC axonal regeneration genes and pathways, namely its scalability.

Finally, one reviewer requested clarification on our comment regarding the dose-dependency of Dlk and of the genetic modifications produced by our CRISPR knockouts. To address this, we have changed some language to clarify the types of mutations we are producing by CRISPR, as well as added further evidence for the dose-dependency of Dlk action (additional panels to Figure 8 of the current submission). We believe that, to those in the know, such a dose-dependency will be surprising and maybe even quite informative.

The additional time and effort to complete the experiments suggested by the reviewers and to revise, clarify, and further explain our thinking has been invaluable to this body of work. We hope you will find the current submission worthy of publication in this journal.

Respectfully,

Nicholas Marsh-Armstrong

Professor

**Point-by-Point: Response to Reviewers**

Below please find (in italics) our point-by-point response to each one of the reviewer comments (in regular type) which we received in response to our initial submission to PNAS.

Reviewer Comments:

Reviewer #1:
Suitable Quality?: Yes
Sufficient General Interest?: No
Conclusions Justified?:No
Clearly Written?:Yes
Procedures Described?:Yes

Comments:
Mechanisms CNS axon regeneration in non-mammalian vertebrates may offer insights relevant for promoting CNS repair in mammals. Nevertheless, systems suitable for genetic screening and in vivo visualization have been sparse. In this study, Fague and Marsh-Armstrong establish a tadpole optic nerve crush (ONC) procedure, demonstrate that ONC does not stimulate significant RGC death in this model, determine that restoration of visual function is primarily dependent on RGC axon regeneration rather than generation of new RGCs, and that RGC axon regeneration, in contrast to primary axon growth, is dependent on the conserved kinase Dlk. Disruption of Dlk is associated, as it is in other species, with suppression of the accumulation of phosphorylated c-Jun in the nucleus, but not with suppression of acute changes in mitochondrial movement, consistent with a role for the transcriptional response in enabling axon regeneration. Overall, the method is innovative and will be of value to the field, and its validation here is well executed. This tadpole model, along with independently developed zebrafish models, should be of substantial utility in deciphering commonalities and differences between the responses of regenerating and non-regenerating species. Given its extraordinary potency in axon regeneration and conservation from worms to flies to both PNS and CNS in mammals, the targeting of Dlk in this study serves as a critical proof-of-principle for this new system. Though generally appealing in its design and execution, the impact of the study is limited by a lack of new insights that might be gained by more directly testing CNS mechanisms downstream of Dlk.

Major comments:
1) A central claim of the manuscript is that Dlk knockout impacts regeneration by virtue of limiting the accumulation of phosphorylated c-Jun in the nucleus rather than by affecting mitochondrial movement. While this is a reasonable inference, it doesn't consider other mechanisms or take advantage of this new system for direct testing of the hypothesis. Though a useful marker of Dlk activation and seemingly quite likely to be of importance here, definitive evidence for a critical role for c-Jun in axon regeneration outside of rodent facial nerve injury is limited, especially in the optic nerve (PMID: 23431164). Analyzing the extent to which knockout of c-Jun recapitulates knockout of Dlk would therefore provide valuable insights into its overall contribution to RGC regeneration.

*We emphatically agreed with this comment regarding direct testing of the role of c-Jun in regeneration and indeed, were already planning for this follow-up experiment following submission of the original manuscript. The results of our experiment, which we report here in this revised manuscript, validated this reviewer’s reservations regarding our conclusions as to the importance of c-Jun in regeneration. Much to our surprise, we find that in fact c-Jun does not appear to be required for RGC axonal regeneration in this system (Figures 8 and S6).*

2) The lack of details on the screen limits the evaluation of this new method. Was Dlk a lone hit amongst a handful of candidates? Or one of several hits amongst hundreds of candidates? Or something in between? Though it is sensible to reveal, validate, and elaborate other hits in independent studies, it would still be of value to provide additional context. Of particular interest would be if the initial screen included additional putative mediators of regeneration. If such candidates do not recapitulate findings in other models, is there evidence to distinguish between limitations of CRISPR in this model, species-specific gene functions, or perhaps differences between CNS and PNS regeneration?

*We agreed with this critique of referring to our new method as a “screen” without elaborating on other targets which we may be working on: with only a single gene reported in the original manuscript this title was unwarranted. Though the reviewer was correct in assuming that we might have screened dozens of other candidates but wished to reveal other hits independently, we have modified our language here to more accurately describe our method as an “assay” rather than a “screen”. We have also included details about a second gene (c-Jun) we have assayed with this new method which is, as per this review comment, another putative regeneration mediator, which does not recapitulate findings in other models and thus points to either species or CNS/PNS differences in regeneration.*

3) It's not certain that immunohistochemistry alone is sufficient to conclude, as definitively as stated here, that "activation of c-Jun post-ONC is completely dependent on Dlk," which "is in contrast to mammalian species." The role of LZK in retrograde signaling to the nucleus in mammals is far less pronounced than that of DLK but was nevertheless initially underappreciated, based on similar types of observations. In part, this is a technical issue, as immunohistochemistry signal is not always linear and may include threshold effects. Also relevant is the evidence that phosphorylation of c-Jun may not be essential for its activation in the context of axon regeneration (PMID: 22372722). Without an examination of the transcriptional response (e.g., RNA-seq, or qRT-PCR of known c-Jun-dependent genes) in the context of Map3k13 disruption, or at least in the context of Dlk knockout, this inference seems to be overstated.

*This reviewer comment was also on point, and we have modified our description to allude to the ambiguities associated with this finding. We have also now included, as per the reference cited, the important note that c-Jun phosphorylation may not be essential for axonal regeneration. Additionally, we have included a new supplemental figure comparing the various different jun variants present in X. laevis to those found in mice and humans, to show both that frogs have clear orthologs for the various mammalian genes (showing that, not surprisingly, Jun functions are likely highly conserved across vertebrates), though intriguingly frogs also a Jun paralog not present in mammals that is conserved in other regenerative species, to be studied as we do a systematic genetic dissection of the relevant MAPK pathway to pinpoint where mammals and frog functionally diverge (in the future) .*

Minor comments:
1) For a broad audience, a discussion of the relative advantages and disadvantages of the tadpole model to zebrafish models would be of value, especially in regards to live imaging and genetic screening capabilities.

*This is a topic near and dear to me, as I did my PhD in zebrafish and indeed years ago submitted an unsuccessful grant application for a multi-species approach incorporating both species. I do believe that our current tadpole model is far superior to anything currently available in zebrafish for an initial moderate to high-throughput screen, but that needs to be followed by secondary less throughput secondary screens (in fish and/or preferably in mice). However, given the new results on cJun and related discussion points, we did not have sufficient space to address the pros and cons of each species for RGC regeneration studies, but will do so in the future after we have scaled-up.*

2) There does not appear to be a callout of Figure 9 in the text.

*All figures in the resubmission are referenced in the text.*

3) Dlk is described in the Results as being upstream of the Janus Kinase (Jak). Might the authors have meant to reference c-Jun N-terminal kinases (Jnk) instead?

*This error has been rectified in the current version.*


Reviewer #2:
Suitable Quality?:No
Sufficient General Interest?:No
Conclusions Justified?:Yes
Clearly Written?:Yes
Procedures Described?:Yes

Comments:
Fague and Marsh-Armstrong established a novel in vivo system in Xenopus to evaluate retinal neuron axon regeneration. The system is highly suitable for in vivo imaging and offers a rapid assay (compared to mammals) to observe optic nerve regeneration. The study was carefully executed. While the study established a new assay, the molecular hit on Dlk was largely characterized in multiple species, including several detailed studies in the mammalian system. Thus the scientific findings have not advanced to a significant level for general readers of PNAS in the fields of visual neuroscience and regenerative medicine. It will be a better fit for a more specialized journal in the developmental biology community.

Major critics include the following -

1) The authors tried to establish an argument that tadpoles are better/faster models for regeneration than adult frogs. This effort is somewhat misleading as this is a highly dynamic developing system. The analogy would be to study neonatal eye regrowth abilities while the eyes develop. The developing system offers higher growth and regrowth potentials. Thus the findings are more relevant for the developmental process and remodeling during developmental injuries and likely yield insights into the fundamental biology of regenerative medicine in the adult central nervous system.

*This critique was quite relevant as there are important differences between tadpoles and adult frogs, as there are between juvenile and adult members of any species. In a new limitations section of the discussion, we make mention of what we believe are the two most relevant differences between our “developing” and adult RGCs, specifically as it relates to the disease where RGC axonal regenerative therapies would be most impactful, glaucoma, limitations that we believe would be largely addressed by additional studies in adult frogs. Such studies in adult frogs are indeed underway, but to be unambiguous they required us creating frogs where the CMZ no-longer add new RGCs, studies that will be reported on in the future. We have also outlined in the Discussion several salient points which we feel explain why we believe this is still a highly relevant, though obviously imperfect, model to deconstruct evolutionarily conserved pathways essential for RGC axonal regeneration, namely its scalability.*

2) The studies are carefully delivered and carefully controlled, employing multiple types of genetic, imaging, and behavioral approaches, reflecting solid training for a graduate thesis. However, the target of choice more or less served as a positive control for a new system. Without other targets /screens (based on CRISPR), the scientific advancement of the new system is yet limited. Suppose the authors did carry a mini/midi CRISPR screen (which is not trivial for an in-vivo Xenopus system, given the complexity of the genome copy numbers). In that case, the study might be of general interest to the broader audience.

*This reviewer comment is quite similar to Major Comment 2 from Reviewer #1 and we have revised our description of our model in the current revision.*

3) The dose-dependent claim in Figure 4: it would be more convincing and reader-friendly if the authors presented a scheme next to the mutations in their claim. The claim on Dlk ∆56-58 (2 copies) was not clear to a non-expert like me - in either the main figure/ associated supplementary text, it would be helpful to illustrate where the mutations were generated and how the catalytic site might be disrupted.

*Figures 3 and 4 of both the original and current description contain details of sequence traces, TIDE indel analysis, and a gene map to illustrate the design, PAM location, and typical mutation (indels) generated from our sgRNAs. Figure 8 of the current submission also incudes another indicator of the dose-dependency of Dlk (and the catalytic sufficiency of the Dlk* ∆*56-58 mutant) in that those animals which carry one or two copies of Dlk* ∆*56-58 do show cJun phosphorylation after ONC, at levels lower than control (WT) animals but higher than animals carrying only full frameshift KOs. While we did not want to overstate the significance of this finding in the manuscript, we do want to point out here how surprised we were to find the dose dependence, as that is something not typically seen in enzymes, once again suggesting that in frogs Dlk is acting through a mechanism that may be distinct from those previously reported.*

4) Related to Point 1 above, while the studies employed multiple novel imaging and behavior approaches, such as those in Figures 5/6, the alternative explanations could be the difference between a developing nervous system vs. an adult nervous system. In other words, the findings sounded interesting and somewhat surprising to present but failed to be generalized to the principles of adult CNS regeneration in general.

*This comment was well-pointed and we have addressed the differences between developing and adult nervous system in the current revision, as well as included more detail on the similarities and differences of X. laevis and mammalian models to further place our results in context of the field at large.*
